# Induction of the alternative lengthening of telomeres pathway by trapping of proteins on *DNA*

**DOI:** 10.1093/nar/gkad150

**Published:** 2023-03-21

**Authors:** Anna M Rose, Tomas Goncalves, Siobhan Cunniffe, Helene E B Geiller, Thomas Kent, Sam Shepherd, Malitha Ratnaweera, Roderick J O’Sullivan, Richard J Gibbons, David Clynes

**Affiliations:** MRC Molecular Haematology Unit, Weatherall Institute of Molecular Medicine, University of Oxford, John Radcliffe Hospital, Oxford OX3 9DS, UK; Department of Paediatrics, University of Oxford, John Radcliffe Hospital, Oxford OX3 9DS, UK; MRC Molecular Haematology Unit, Weatherall Institute of Molecular Medicine, University of Oxford, John Radcliffe Hospital, Oxford OX3 9DS, UK; Department of Oncology, University of Oxford, Oxford OX3 7DQ, UK; Department of Oncology, University of Oxford, Oxford OX3 7DQ, UK; MRC Molecular Haematology Unit, Weatherall Institute of Molecular Medicine, University of Oxford, John Radcliffe Hospital, Oxford OX3 9DS, UK; Department of Oncology, University of Oxford, Oxford OX3 7DQ, UK; Department of Oncology, University of Oxford, Oxford OX3 7DQ, UK; Department of Pharmacology and Chemical Biology, UPMC Hillman Cancer Center, University of Pittsburgh, Pittsburgh, PA, USA; MRC Molecular Haematology Unit, Weatherall Institute of Molecular Medicine, University of Oxford, John Radcliffe Hospital, Oxford OX3 9DS, UK; Department of Oncology, University of Oxford, Oxford OX3 7DQ, UK

## Abstract

Telomere maintenance is a hallmark of malignant cells and allows cancers to divide indefinitely. In some cancers, this is achieved through the alternative lengthening of telomeres (ALT) pathway. Whilst loss of ATRX is a near universal feature of ALT-cancers, it is insufficient in isolation. As such, other cellular events must be necessary - but the exact nature of the secondary events has remained elusive. Here, we report that trapping of proteins (such as TOP1, TOP2A and PARP1) on DNA leads to ALT induction in cells lacking ATRX. We demonstrate that protein-trapping chemotherapeutic agents, such as etoposide, camptothecin and talazoparib, induce ALT markers specifically in ATRX-null cells. Further, we show that treatment with G4-stabilising drugs cause an increase in trapped TOP2A levels which leads to ALT induction in ATRX-null cells. This process is MUS81-endonuclease and break-induced replication dependent, suggesting that protein trapping leads to replication fork stalling, with these forks being aberrantly processed in the absence of ATRX. Finally, we show ALT-positive cells harbour a higher load of genome-wide trapped proteins, such as TOP1, and knockdown of TOP1 reduced ALT activity. Taken together, these findings suggest that protein trapping is a fundamental driving force behind ALT-biology in ATRX-deficient malignancies.

## Graphical Abstract

**Graphical Abstract:**
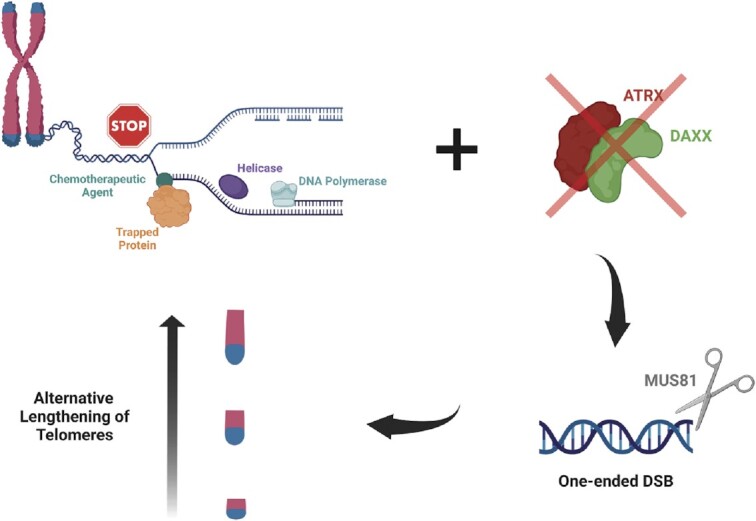
In the context of ATRX/DAXX loss, chemotherapeutic agents which trap proteins on DNA lead to replication fork collapse, MUS81 cleavage and induction of the ALT pathway (Created with Biorender.com).

## INTRODUCTION

Due to the inherent inability of DNA polymerases to replicate the distal ends of linear chromosomes, chromosomal DNA is progressively shortened with each round of cell division. To circumvent potentially detrimental effects on genome stability and loss of genetic information, the genome has developed specialised nucleoprotein structures, telomeres, which are comprised of many kilobases (kb) of a tandem repeat sequence, TTAGGG, culminating in a 3’ protrusion of single stranded G-rich DNA of 50–400 nucleotides. Telomeric sequence is bound by a specialised protein complex, denoted Shelterin, which comprises the proteins TRF1, TRF2, POT1, TIN1, TPP1 and RAP1 (1). Telomeres range from 3 to 12 kb in humans and progressively shorten by about 200 bp per cell division due to the end-replication problem. Once telomeres reach a critical length, termed the Hayflick limit, they elicit DNA damage checkpoint activation, leading to cellular senescence or telomere-induced apoptosis.

One hallmark of cancer cells is their ability to circumvent telomere shortening via a telomere maintenance mechanism (TMM). In the majority of cancers, this is achieved through upregulation of telomerase, a specialised ribonucleoprotein that acts to progressively add telomeric repeats to the end of chromosomes. More recently it has emerged that a subset of cancers maintain their telomere length through a telomerase independent TMM, known as the alternative lengthening of telomeres (ALT) pathway. The ALT pathway is particularly prevalent in cancers of mesenchymal origin (such as osteosarcoma), and several cancers of the central nervous system, such as glioblastoma (2). ALT in human cancer cells is generally considered to be a form of aberrant telomere recombination and conservative DNA synthesis, known as break-induced replication (BIR), occurring during both the G2 and mitosis phases of the cell cycle (3–5). A three-protein axis has been implicated in facilitating ALT-mediated telomere synthesis; POLD3, PCNA and RAD52 (3–5). A second RAD52-independent pathway has also recently been reported, suggesting that ALT is in fact a bifurcated pathway, with similarities to telomerase independent telomere maintenance pathways originally described in budding yeast (6,7).

Further work in human cells has suggested that replication stress arising at telomeres can potentiate the ALT pathway (4). Indeed, owing to their repetitive nature, telomeres are thought to be inherently difficult sequences to replicate and telomeres have been shown to phenotypically resemble common fragile sites due to the overt fragility they exhibit under conditions of replication stress (8). This is likely in large part due to the propensity of the G-rich repetitive telomeric sequence to adopt non-canonical DNA secondary structures, including the G-quadruplex (G4) conformation and R-loops; three-stranded nucleic acid structures consisting of an RNA:DNA hybrid and a displaced piece of single stranded DNA (9). In line with this notion, abrogation of FANCM activity, which has known roles in R-loop resolution and fork stabilisation (10–12), or depletion of RNase H, which can degrade DNA:RNA hybrids, have both recently been shown to potentiate markers of the ALT pathway (12–14).

Almost all ALT+ cancer cells exhibit loss of the *ATRX* gene and/or its interaction partner *DAXX* (15–17), however, the precise role of ATRX/DAXX loss in the initiation and maintenance of ALT remains unclear. Previous work by our lab and others has demonstrated that ectopic expression of ATRX leads to a DAXX-dependent suppression of the ALT pathway (18,19). However, depletion or knockout of *ATRX* in telomerase positive or primary cell lines is generally insufficient to induce markers of the ALT pathway (18–21), with the notable exception of a minority of glioma cell lines (22–24). ALT has also been induced in cells where further proteins, along with ATRX, have also been knocked down; for example, p53, TERT and the histone demethylase KDM4B (25). There must, therefore, be other genetic, epigenetic or cellular events required for ALT induction that act in concert with ATRX loss – but, to date, what these events are remains unclear.

ATRX is a multifunctional protein, involved in various critical cellular and genetic processes. ATRX is a chromatin remodelling factor of the Snf2 family which, together with the histone chaperone DAXX, facilitates the incorporation of the histone variant H3.3 into defined genomic sites, such as pericentric and telomeric chromatin (26–28). ATRX has various roles in the maintenance of genome stability, including regulation of non-canonical DNA secondary structures, such as G4s and R-loops, with ATRX null cells displaying increases in both of these structures (29–32). ATRX also has multiple reported roles in DNA replication, including the prevention of replication fork stalling, potentiation of fork restart and the prevention of excessive nucleolytic degradation of stalled forks (20,31,33–35). Additionally, ATRX facilitates DNA double strand break (DSB) repair, through both homologous recombination (HR) and non-homologous end joining (NHEJ) pathway (36–39). It is clear that the loss of one or more of these roles could be instrumental to the induction of the ALT pathway – but the exact nature of the perturbation causing the ‘second factor’ remains undefined.

In this work, we explore the role of protein trapping in ATRX-deficient cells and demonstrate that formation of DNA-protein covalent complexes (DPCCs) and/or accumulation of proteins on DNA is a fundamental driving force in both natural and artificial models of ALT-cancer. We hypothesise that these DPCCs cause replication fork collapse in the absence of ATRX, which is the substrate for subsequent BIR and ALT activity.

## MATERIALS AND METHODS

### Cell lines and cell culture conditions

All cell lines were obtained from ATCC with the exception of HeLa LT which was a gift from Roderick O’Sullivan (University of Pittsburgh, USA), as described in (21). All cells were cultured at 37°C in 5% CO_2_ in standard Dulbecco's modified Eagle's medium (DMEM) media supplemented with 10% foetal calf serum, 1% l-glutamine and 1% PenStrep (all Gibco). Cells were split every 2–4 days to maintain at a confluency no greater than 90% using 0.05% trypsin/EDTA (Gibco).

### CRISPR-Cas9 knockout

CRISPR-Cas9 knockout of *ATRX* was performed using a modified pSpCas9(bb)-2A-GFP tagged Cas9 vector containing sgRNAs targeting exon 16 of ATRX (TOP: 5’-caccGTCCAATAACAACCA^AGT-3’, BOTTOM: 5’-aaacACT^TGGTTGTTATTGGAC-3’) with an expected cut site at lysine 1536. Cells were sorted on a BD FACSAria Fusion cell sorter based on GFP expression 24 h after transfection into single wells and grown into clones. Knockout was determined by western blotting. DAXX knockouts were performed using a commercially available DAXX Cas9 plasmid (Santa Cruz, sc-400686-knockout-2) and were FACS sorted based on GFP expression. Transfected cells were selected using 0.4 μg/ml puromycin and then sorted into single wells to obtain clones. Combinatorial knockouts were made through sequential knockout of genes.

### Treatment of cells with genotoxic agents

Cells were treated with these genotoxic agents at the following doses for 24–72 h prior to downstream analysis with the ALT assays used in this study: pyridostatin/PDS (Sigma) 5 μM; camptothecin/CPT (Sigma) 50 nM; etoposide/ETO (Sigma) 0.5 μM; PJ34 (Calbiochem) 5 μM; veliparib (Stratech) 5 μM; olaparib (Selleckchem) 5 μM; niraparib (Stratech) 5 μM; talazoparib (Selleckchem) 5 μM; CX-5461 (Sigma) 1 μM; hydroxyurea/HU (Sigma) 50–400 μM and aphidicolin/APH (Sigma) 0.2–0.4 μM. Formaldehyde (500 μM, Sigma) was added to cells for one hour, then media was changed and downstream analysis performed at 48–72 h, as with other agents. Negative controls with the drug diluent (water, PBS or DMSO) but without drug were included with each assay.

### Western blotting

To prepare whole cell lysates, about 2 × 10^6^ cells were lysed in 200 μl ice-cold IgePal lysis buffer (50 mM Tris–HCl pH 7.4, 100 mM NaCl, 1 mM MgCl_2_, 10% glycerol, 5 mM NaF, 0.2% IgePal-CA630) containing Pierce protease inhibitor tablet (Thermo Fisher Scientific) for 45 min on ice. Insoluble components were removed by centrifugation and protein lysate concentration was calculated by NanoDrop. At least 10 μg of lysate was loaded per lane into precast 4–12% bis–tris gels with MOPS running buffer (both Thermo Fisher Scientific). Membranes were transferred at 4°C overnight onto 0.45 μm pore size PVDF membranes (Millipore) pre-activated in 100% methanol using NuPage transfer buffer (Thermo Fisher Scientific) supplemented with 10% methanol. The following day, membranes were rinsed briefly in PBST (0.1% tween in PBS) and then blocked for 1 hour at room temperature in 5% milk in PBST. Primary antibody incubations then followed in 2.5% milk in PBST. Membranes were washed three times for 10 min with PBST before incubation with HRP-conjugated secondary antibodies diluted in 2.5% milk in PBST for 1 h at room temperature. Membranes were once again washed three times for 10 min with PBST and membranes were then developed using SignalFire ECL reagent (Cell Signaling) onto X-ray films (Amersham).

To prepare subcellular fractions of nuclear soluble and chromatin bound fractions, 1 million HeLa LT ATRXΔ1 cells were treated with the panel of PARPi drugs for 48 h and then cells were collected. For the fractionation, a subcellular protein fractionation kit (78840, Thermo Fisher Scientific) was used according to manufacturer's instructions. Immunoblotting was carried out as above. Signal intensity was measured using ImageJ software.

The following primary and secondary antibodies were used with the indicated dilutions: mouse anti-PARP1 (Santa Cruz, sc-8007, 1:500), mouse anti-H3 (BioLegend, 819414, 1:100 000), rabbit anti-ATRX (Abcam, ab97508, 1:1000), mouse anti-ATRX (39F, 1:17) (40), mouse anti-alpha Tubulin (Abcam, ab7291, 1:50 000), mouse anti-BLM (Santa Cruz, sc-365753, 1:200), mouse anti-POLD3 (Novus Biologicals, H00010714-M01, 1:500), mouse anti-RAD52 (Santa Cruz, sc-365341, 1:200), rabbit anti-DAXX (Sigma, D7810, 1:2000), mouse anti-TOP2A (Santa Cruz, sc-365916, 1:200), rabbit anti-GAPDH (Cell Signaling, 2118, 1:1000), mouse anti-TOP1 (Santa Cruz, sc-32736, 1:500), mouse anti-pADPr (Santa Cruz, sc-56198, 1:100), mouse anti-PARP1 (Santa Cruz, sc-8007, 1:200), mouse anti-MUS81 (Santa Cruz, sc-376661, 1:200), mouse anti- -βActin − Peroxidase (Sigma-Aldrich, A3854, 1:20000), mouse anti-phospho-H2AX-S139 (Sigma-Aldrich, 05–636-I, 1:2000), rabbit anti-ASF1a (Cell Signaling, 2990S, 1:1000), rabbit anti-mouse IgG HRP (Sigma, A9044, 1:5000), goat anti-rabbit IgG HRP (Thermo Fisher Scientific, 31460, 1:5000).

### siRNA knockdowns

siRNA experiments were performed using commercially available ON-TARGETplus siRNA SmartPools (Dharmacon) targeting BLM (L-007287-00-0005), POLD3 (L-026692-01-0005), RAD52 (L-011760–00-0005), ASF1A (L-020222-02-0005), ASF1B (L-020553-00-0005), TOP1 (L-005278–00-0005), TOP2A (L-004239-00-0005), PARP1 (L-006656-03-0005) and MUS81 (L-016143-01-0005) or a non-targeting control pool (D-001810-01-05). Cells were reverse transfected when seeding cells to wells or coverslips using Lipofectamine RNAiMAX (Thermo Fisher Scientific) to a final concentration of 5 pmol following the manufacturer's instructions. For CC assay and Western blotting analysis, 300 000 cells were seeded out into 6 cm culture dishes with 1.25 ml DMEM and 1.25 ml transfection mix (5 μl RNAiMAX reagent and 10 nM of siRNA diluted in Opti-MEM (Gibco)). Transfections were scaled down accordingly for immunofluorescence analysis; 50 000 cells were seeded out into 24-well plates with 13 mm coverslips with 125 μl DMEM and 125 μl transfection mix (0.4 μl RNAiMAX reagent and 10 nM of siRNA diluted in Opti-MEM. 48 h after transfection, cells were forward transfected to ensure effective knockdown. 72 h after transfection, cells were treated with genotoxic agents and incubated for a further 48 h before harvesting and downstream processing (DNA extraction, protein extraction and immunofluorescence). Western blots were performed to check efficiency of knockdown. A negative control, using a scrambled siRNA sequence, was included with each assay.

### shRNA knockdowns

shRNA experiments were performed using the following commercially available MISSION shRNA plasmid DNA: MISSION shRNA TOP1 (NM_003286-3990) and MISSION shRNA PARP1 (NM_001618-7930) (both Sigma) that were packaged into lentiviruses in house. Puromycin kill curves were performed to establish the minimum lethal dose for untransduced cells at 48h timepoint. Cells (30,000) were seeded into a 24-well plate and, the following day, 250 μl of the lentivirus mix (containing 35 μl of lentivirally packaged shRNA, 215 μl of DMEM and 0.3 μl of polybrene) was added. Seventy-two h after transduction, cells were held under selection with puromycin (3 μg/ml) for up to 8 days before being harvested or fixed for downstream assays. Western blots were performed to check efficiency of knockdown. A negative control, using a scrambled shRNA sequence, was included with each assay.

### Immunofluorescence/ImmunoFISH

Immunofluorescence experiments were performed with 50 000 cells seeded onto 13 mm #00 thickness glass coverslips in 24-well tissue culture plates. Cells were briefly washed once in PBS, pre-permeabilised in 0.5% triton X-100 (Sigma) in PBS for 1 minute on ice and then fixed with 4% paraformaldehyde (Thermo Fisher Scientific; 16% diluted to 4% in PBS) for 20 min at room temperature. Fixed cells were then washed three times in PBS for 5 min and then permeabilised with 0.5% triton X-100 on ice for 6 min. The cells were washed a further three times in PBS for 5 min each and then blocked for 1 h in blocking solution (1% BSA in PBS). After blocking, samples were incubated for at least 1 h with primary antibodies diluted in blocking buffer. Samples were washed four times with PBST for 5 min and then incubated for 1 h with fluorescently labelled secondary antibodies raised in the appropriate species diluted in blocking buffer. Following three more 5 minute washes with PBST, the samples were mounted in VectaShield containing DAPI and visualised using a DeltaVision widefield microscope with either 60× or 100× objectives. Images were processed using Fiji ImageJ, and downstream analysis of co-localisation and/or foci number and intensity was performed on CellProfiler (41–43). Cells from at least two independent biological replicates were analysed for each experimental condition, with a minimum of 100 cells per repeat. The following primary and secondary antibodies were used with the indicated dilutions: mouse anti-PML (Santa Cruz, sc-966, 1:300), rabbit anti-TRF2 (Novus Biologicals, NB110-57130, 1:500), mouse anti-RPA32 (Abcam, ab2175, 1:500), rabbit anti-ATRX (Abcam, ab97508, 1:500), mouse anti-pATM[S1981] (Santa Cruz, sc-47739, 1:250), mouse anti-BLM (Santa Cruz, sc-365753, 1:300), mouse anti-Top1cc (Merck, MABE1084, 1:200), goat anti-rabbit Alexa Fluor 568 (Life Technologies, A11036, 1:3000), goat anti-mouse Alexa Fluor 488 (Life Technologies, A11029, 1:3000). For Top1cc immunofluorescence, pre-permeabilisation time was increased to 5 min and permeabilisation time to 20 min.

ImmunoFISH experiments were carried out as described above up until the PBST washes after secondary antibody incubation. Following these, samples were post-fixed with 4% paraformaldehyde (as above) for 10 min at room temperature. If denatured telomeres were required (all experiments except RPA-ssTel analysis), 3.5 N HCl was added to the coverslips at room temperature for 13 min and the reaction was then quenched by washing twice for 5 min with ice cold PBS. Samples were then washed twice with PBST for 5 min and once with 2× SSC buffer (0.3 M sodium chloride, 0.03 M sodium citrate, pH 7). A Cy3-[CCCTAA]_5_ probe was then hybridised onto the coverslip overnight at 37°C in a humidified chamber by placing the coverslips cell side face down onto 10 μl of probe hybridisation mix (1 μl of 10 μM Cy3-[CCCTAA]_5_ probe per 49 μl hybridisation buffer (25% formamide, 2× SSC, 200 ng/μl salmon sperm, 5× Denhardt's solution, 50 mM phosphate buffer, 1 mM EDTA)) dispensed on parafilm. The following day, coverslips were returned to 24-well dishes and washed three times for 10 min each with 2× SSC at 37°C, followed by two 5 min washed in PBST and one 5 min wash in PBS. Coverslips were then mounted with DAPI and imaged as above.

### Metaphase spreads and telo-FISH

Cells were seeded out into 6 cm dishes and treated with PDS for 24 h. Karyomax colcemid (50 μl of 10 μg/ml, Gibco) was added to the cells for 1–4 h to arrest cells at the metaphase stage of mitosis. Cells were then trypsinised and collected. Pre-warmed hypotonic solution (75 mM KCl) was then added to the cells and incubated for 15 min at 37°C. Cells were spun down and fixative (3:1 methanol:glacial acetic acid) was gently added dropwise to the pellet to a final volume of 10 ml. Cells were incubated at –20°C for 30 min, spun down, and fixative was replaced twice. Cells were then resuspended in an appropriate volume of fresh fixative and cells were dropped onto warmed glass slides. Spreads were hardened for 5 days at room temperature and then Telo-FISH was carried out as above. Fragile telomeres were scored when multiple telomeric signals were seen that were spatially separated from chromatid ends, as in (8).

### C-circle assay

Genomic DNA was extracted from pellets of up to 1 × 10^6^ cells using the PureLink genomic DNA extraction kit (Thermo Fisher Scientific) and quantified using a NanoDrop. 30 ng of genomic DNA was amplified in a 20 μl rolling circle amplification reaction containing 7.5 U of φ29 polymerase (New England Biolabs), 0.1% Tween-20, 200 μg/ml BSA (New England Biolabs) and 1 mM each of dTTP, dGTP and dATP (all New England Biolabs) diluted in nuclease-free water. The reactions were incubated in a PCR cycler (Bio-Rad T100 Thermal Cycler) for 8 h at 30°C followed by 20 min at 65°C. A negative control (without φ29 polymerase) and a positive control (U2OS gDNA) was included in each run. Amplified samples were then diluted with 180 μl 2× SSC buffer and transferred onto a Zeta-Probe membrane (Bio-Rad) using a slot blot filtration manifold (Bio-Rad BIO-DOT, 48-well). The membranes were dried for 15 min at room temperature and then UV crosslinked using the ‘Auto Crosslink’ setting on a Stratalinker 2000 (UV-A). The membrane was pre-hybridised with 10 ml DIG Easy Hyb (Roche) for 20 min at room temperature on a roller and then incubated in a 37°C hybridisation oven for 2 h with a 3’DIG-labelled [CCCTAA]_5_ probe diluted in 10 ml DIG Easy Hyb to a final concentration of 40 nM. Membranes were then washed twice for 5 min in MS wash buffer (0.1 M maleic acid, 3 M NaCl, 0.3% Tween-20, pH 7.5) and blocked in MS blocking buffer (1% milk and 1% BSA in 0.1 M maleic acid, 3 M NaCl, pH 7.5) for 30 min at room temperature. Anti-DIG-AP Fab fragments (Roche) were added to the MS blocking buffer (1:20 000) and the membranes were incubated for 30 min at room temperature. The membranes were washed three times for 15 min with MS wash buffer and then incubated with 2 ml of CDP-Star chemiluminescent substrate solution (Roche) for X-ray film detection. Intensities on membrane images were quantified using ImageJ software. Unless otherwise stated, normalised CC levels (A.U) were expressed relative to a U2OS reference sample (30 ng) from the same membrane. A gDNA dilution series (4:2:1) was loaded onto each membrane to assess assay linearity. Assays were performed on at least two independent DNA samples for each test condition.

### Terminal restriction fragment (TRF) assay

Genomic DNA (>2 μg) was digested overnight at 37°C with *HinfI* and *RsaI* restriction endonucleases (NEB). Following digestion, samples were run on 0.8% agarose gels in 1× TAE at 60 V overnight. Gels were denatured using 1 M NaOH and then neutralised with neutralisation buffer (0.5 M Tris–HCl, 1.5 M NaCl) and blotted onto Zeta-Probe membrane by upward capillary transfer overnight. Blots were then probed with a DIG-tagged telomere probe as described above. The blots were analysed and quantified using TeloMetric software (44).

### Monochrome multiplex qPCR (mm-qPCR)

MM-qPCR was carried out as previously reported with minor alterations (45). Primers were bought through Life Technologies using standard desalted oligonucleotides. Primer sets used were GlobinF (CGGCGGCGGGCGGC-GCGGGCTGGGCGGCTTCATCCACGTTCACCTTG), GlobinR (GCCCGGCCCGCCGCGCCCGTCCCGCCG-GAGGAGAAGTCTGCCGTT), hTeloG (ACACTAA-GGTTTGGGTTTGGGTTTGGGTTTGGGTTAGTGT) and hTeloC (TGTTAGGTATCCCTATCCCTATCCCTATCCCTATCCCTAACA). Five concentrations of reference genomic DNA purified from HeLa LT were prepared by 3-fold serial dilution (from 150 ng to 1.85 ng) to generate standard curves for relative quantitation of T/S ratios. For each sample, 20 ng of genomic DNA was mixed with 0.75× PowerUp SYBR Green Master Mix (Thermo Fisher Scientific), the relevant forward and reverse primers (300 nM), and water to a final volume of 20 μl per well and analysed using a Thermo Fisher QuantStudio 3 qPCR machine with the following cycle conditions: denaturation for 15 min at 95°C, followed by two cycles of 15 s at 94°C/15 s at 49°C and 32 cycles of 15 s at 94°C/10 s at 62°C/15 s at 74°C with signal acquisition and 10 s at 84°C/15 s at 88°C with signal acquisition. Samples were run in triplicate, and analysis was repeated six times using independent runs.

### Reverse transcription quantitative polymerase chain reaction (RT-qPCR)

RNA was extracted from cells using the PureLink RNA mini kit (Thermo Fisher Scientific). 1 μg of total RNA was reverse transcribed to cDNA using 200 U of SuperScript IV reverse transcriptase (Thermo Scientific), and 50 μM random hexamer oligos, according to manufacturer instructions. cDNA was diluted to 200 μl with nuclease-free water. For each sample, 5 μl of cDNA was mixed with 0.75x PowerUp SYBR Green Master Mix (Thermo Fisher Scientific), the primers (300 nM) and water to a final volume of 20 μl per well and analysed using a Thermo Fisher QuantStudio 3 qPCR machine with the following cycle conditions: 95°C for 15 min, followed by 40 cycles of 10s at 95°C, 20s at 60°C and 20s at 72°C. Melting curve analysis was performed immediately following this. Primers were bought through Life Technologies using standard desalted oligonucleotides. Primers used were hTERT F1579 (GCTGACGTGGAAGATGAGCGTGC) hTERT R1616 (TCCTCACGCAGACGGTGCTCTG), 7SK F7 (GAGGGCGATCTGGCTGCGACAT) and 7SK R112 (ACATGGAGCGGTGAGGGAGGAA). *TERT* expression was normalised to *7SK* expression using the ΔCT method. Data is expressed as ΔΔCT comparing the untreated controls versus PDS-treated cells. Averages were calculated from two biological replicates, each run in triplicate.

### RADAR (rapid approach to DNA adduct recovery) assay

RADAR assay was performed as in (46) with minor modifications. Approximately 5 × 10^5^ cells were treated with PDS, CPT, ETO or CX-5461 (as above) for 1 h. After drug treatment, medium was aspirated and cells were lysed directly on the plate by adding 1 ml of lysis reagent (RLT Plus, Qiagen). Nucleic acids were recovered by adding 0.5× volume of 100% ethanol, incubation at -20°C for 10 min and then centrifugation at maximum speed at 4°C for 15 min. The supernatant was removed and the pellet was washed twice with 75% ethanol, followed by 10 min centrifugation at maximum speed. The nucleic acid pellet was then dissolved in 200 μl of freshly prepared 8 mM NaOH and rotated overnight at 4°C to ensure complete solubilisation.

Samples were then slot blotted onto PVDF membranes or Zeta-probe membranes which were then probed for using standard Western blotting as above. Antibodies used were mouse anti-TOP1 (Santa Cruz, sc-32736, 1:200), mouse anti-TOP2A (Santa Cruz, sc-365916, 1:200), mouse anti-dsDNA (Abcam, ab27156, 1:10 000).

### Quantification and statistical analysis

Statistical analysis was done using GraphPad Prism 9 (GraphPad Software Inc.) and Social Science Statistics calculators (https://www.socscistatistics.com/). Unpaired t-tests were used to compare two groups and one-way ANOVA with Welch correction was used to compare more than two groups (both parametric data). Kruskall-Wallis test was used for non-parametric unpaired data. Chi-squared test was used for 2 × 2 contingency analysis. Linear regression was used for correlation analysis. Sample sizes and *P*-values are shown in the figure legends and significance was considered as * P < 0.05. ** *P* < 0.01, *** *P* < 0.001, **** *P* < 0.0001. ns denotes no significance.

## RESULTS

### Stabilisation of G-quadruplex structures induces an ALT-phenotype in ATRX-null cells

Owing to their highly repetitive nature, telomeric sequences have a high propensity to adopt non-canonical DNA secondary structures, including the G-quadruplex (G4) conformations and R-loops (9). Recent work has shown that telomeres of ALT cells are characteristically enriched in both G4 structures and R-loops and form a linked structure known as a G-loop, where a G4 and an R-loop form on opposing strands (47). Based on these findings, we investigated whether stabilisation of G4 structures can induce ALT.

**Figure 1.**
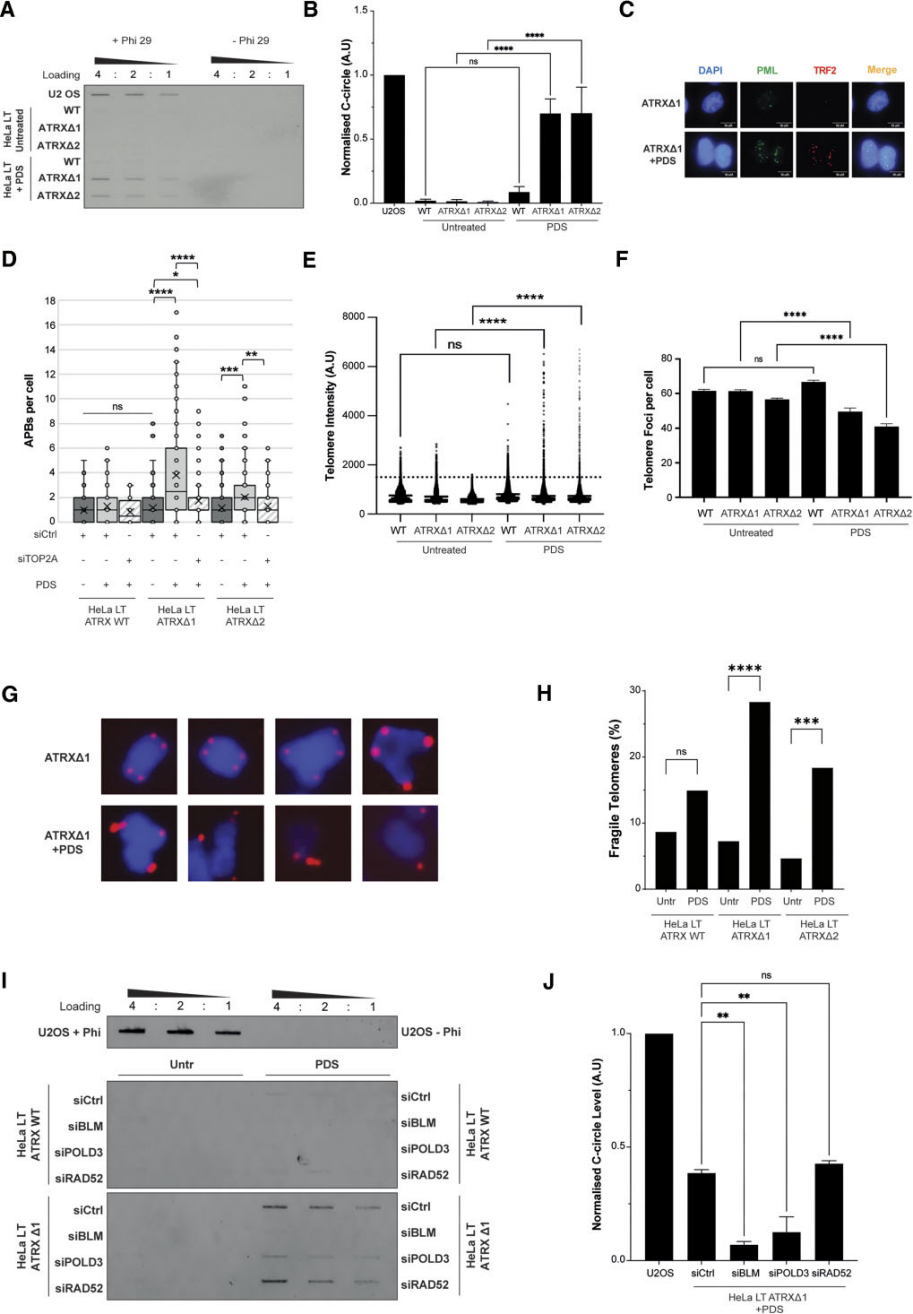
Treatment with PDS in combination with ATRX loss triggers ALT markers. (**A**) Representative C-circle blot showing C-circle accumulation specifically in ATRX knockout clones following 48h treatment with 5 μM PDS. The left three columns are reactions in the presence of phi polymerase while the right three columns are a no phi polymerase negative control. Ratios indicate the samples were loaded in two-fold serial dilutions. U2OS is loaded as a positive control. (**B**) Quantification of (A), three biological replicates run in triplicate. **** *P*< 0.0001, one-way ANOVA with Welch correction. (**C**) Representative images of APB induction in HeLa LT ATRX knockout clones upon 48h treatment with 5 μM PDS. (**D**) Quantification of (C), with and without siTOP2A knockdown, >200 nuclei analysed across 3 biological replicates. * *P*< 0.05, ** *P*< 0.01, *** *P*< 0.001, **** *P*< 0.0001, Kruskall–Wallis test. (**E**) Telomere intensity in HeLa LT ATRX knockout clones upon 48h treatment with 5 μM PDS. >200 nuclei analysed across three biological replicates. Foci considered intense are shown above dotted line 1500 A.U. **** *P*< 0.0001, one-way ANOVA with Welch correction. (**F**) Telomere foci per nuclei in HeLa LT ATRX knockout clones upon 48 h treatment with 5 μM PDS, >200 nuclei analysed across three biological replicates. **** *P*< 0.0001, one-way ANOVA with Welch correction. (**G**) Representative images of metaphase spreads stained with a telomere FISH probe in untreated and 5 μM PDS treated cells for 24 h. (**H**) Quantification of (G). Fragile telomeres were scored when multiple signals were seen that were spatially separated from chromatid ends. >100 metaphases analysed across two biological replicates. *** *P*< 0.001, **** *P*< 0.0001, chi-squared test from 2 × 2 contingency. (**I**) Representative C-circle blot showing that knockdown of BIR machinery prevents induction of C-circles in ATRX knockout cells treated with 5 μM PDS. (**J**) Quantification of (I), two biological replicates run in triplicate. ** *P*< 0.01, one-way ANOVA with Welch correction.

The HeLa long telomere cell line (HeLa LT) is a HeLa subclone which has previously been shown to be amenable to ALT induction through depletion of ASF1 (21). Two independent *ATRX* CRISPR-Cas9 knockout HeLa LT clones were generated (Supplementary Figure S1A) and, consistent with previous reports, neither clone elicited an increase in any of the cardinal ALT markers, including C-circles (Supplementary Figures S1B and S1C), ALT-associated PML nuclear bodies (APBs) (Supplementary Figures S1D and E) or telomere length heterogeneity (Supplementary Figure S1F). Strikingly, however, addition of a G4 stabilising ligand, pyridostatin (PDS), resulted in increased levels of each of these key ALT markers in the two clones lacking ATRX (but not wildtype cells) – and the level was comparable to the archetypical ALT cell line, U2OS (Figures 1A–D). Addition of PDS also elicited an increase in telomere intensity and a decrease in total telomere number, this being consistent with increased telomere clustering and synthesis as observed in ALT (Figures 1E and F). We also saw an increase in telomere heterogeneity (Supplementary Figures S2A and B), an increase in telomere copy number as measured by qPCR (Supplementary Figure S2C) and repression of TERT expression (Supplementary Figure S2D). These data were strongly indicative of a *bona fide* switch to ALT-based telomere maintenance.

Another hallmark of ALT activity is increased replication stress and accumulation of DNA damage signals at telomeres, termed telomere damage induced foci (TIFs), a potential driver of ALT (16). Telomeric accumulation of DNA damage markers (such as 53BP1, γH2AX and ATM phosphorylated at serine 1981 (pATM^s1981^)) have all been used to identify TIFs (48). Immuno-FISH analysis assessed co-localisation between pATM^S1981^ and telomeric foci, and allowed us to quantify levels of DNA damage at telomeres. Following treatment with PDS, both ATRX wildtype and deficient cells showed increased levels of telomeric pATM^S1981^, however, ATRX deficient cells had significantly higher TIF levels (Supplementary Figure S3). We also measured telomere fragility in our cells, finding that ATRX-null cells treated with PDS had a significantly higher percentage of fragile telomeres as compared to untreated cells (Figures 1G and H). It is generally considered that fragile telomeres result from aberrant processing of stalled replication forks and, as such, can be used as a measure of replication fork stalling (8).

Previous work has shown that the human ALT pathway relies on BIR-dependent mechanisms of telomere synthesis. The BIR pathway is a three-protein axis comprised of POLD3, PCNA and RAD52 (3–5). BLM helicase has also been shown to be the downstream effector in the BIR axis and is vital for APB assembly with subsequent ALT telomere synthesis (7,21,49). To confirm that the observed induction of ALT in our system was a BIR-dependent process, we knocked down BLM, POLD3 and RAD52 in our cells in untreated and PDS treated cells (Supplementary Figure S4A). We found that knockdown of BLM and POLD3 significantly reduced the induction of C-circles in our ATRX knockout cells treated with PDS (Figures 1I and J). APB induction was also prevented when BLM was knocked down (Supplementary Figures S4B and C). RAD52 knockdown, however, failed to reduce C-circle levels (Figures 1I and J), supporting previous work suggesting that C-circle formation is RAD52-independent (7). We also confirmed that BLM is recruited to telomeres upon PDS treatment, and this recruitment is significantly greater in ATRX knockout cells *versus* wildtype (Supplementary Figures S4D and E).

ATRX is recruited to GC-rich and repetitive regions of the genome—such as telomeres—where it has been shown to have a role in unfolding of G4-structures at these genomic regions (30–32,50). We therefore examined the recruitment of ATRX to telomeres following PDS treatment. Immuno-FISH analysis demonstrated an increased number of ATRX foci per nucleus and a strong co-localisation of ATRX protein at telomeres following exposure to the drug, supporting the previously described role for ATRX in the resolution of G4s (Supplementary Figures S4F–H). Taken together, these data confirm that following treatment with the G4-stabilising agent PDS, there is recruitment of ATRX to telomeres and, in the absence the ATRX, telomeres undergo aberrant processing via a BIR-dependent process resulting in markers of the ALT pathway.

As a small minority of ALT-cancer cells exhibit DAXX loss, rather than ATRX loss, we generated CRISPR-Cas9 mediated DAXX knockout clones in the HeLa LT cell line, both in the context of wildtype ATRX and ATRX knockout (Supplementary Figure S5A). On treatment with PDS, markers of ALT were observed in the DAXX-depleted clones as were previously seen in the ATRX-depleted clones (Supplementary Figures S5B–D). Importantly, co-depletion of both ATRX and DAXX failed to confer any cumulative increase in cardinal ALT markers, supporting an epistatic relationship between the two proteins and implicating the H3.3 deposition pathway in the prevention of ALT.

ALT has been suggested to be associated with telomeric replicative stress, with an accumulation of RPA2 at telomeres upon ALT induction (21,51) and an increase in single stranded telomeric DNA (ssTel), as detected by non-denaturing telomeric FISH (52). Addition of PDS significantly increased detectable RPA-ssTel foci in both ATRX knockout clones, with no detectable increase in wildtype ATRX HeLa LT cells (Supplementary Figures S5E and F). Similarly, an increase in RPA-ssTel foci was seen in the DAXX knockout clones (Supplementary Figure S5G).

### Trapping of TOP2A protein leads to induction of ALT

It has been suggested that stable non-canonical DNA structures, such as G4s, promote the formation of topoisomerase 2 (TOP2)-mediated DNA breaks (53). The cytotoxic effect of G4 stabilizing drugs (such as PDS and CX-5461) has been shown to be dependent on trapping of TOP2A and presence of R-loop structures (54,55). This led us to consider whether the observed induction of ALT upon PDS treatment of *ATRX*-null HeLa LT cells is dependent on TOP2A trapping.

**Figure 2.**
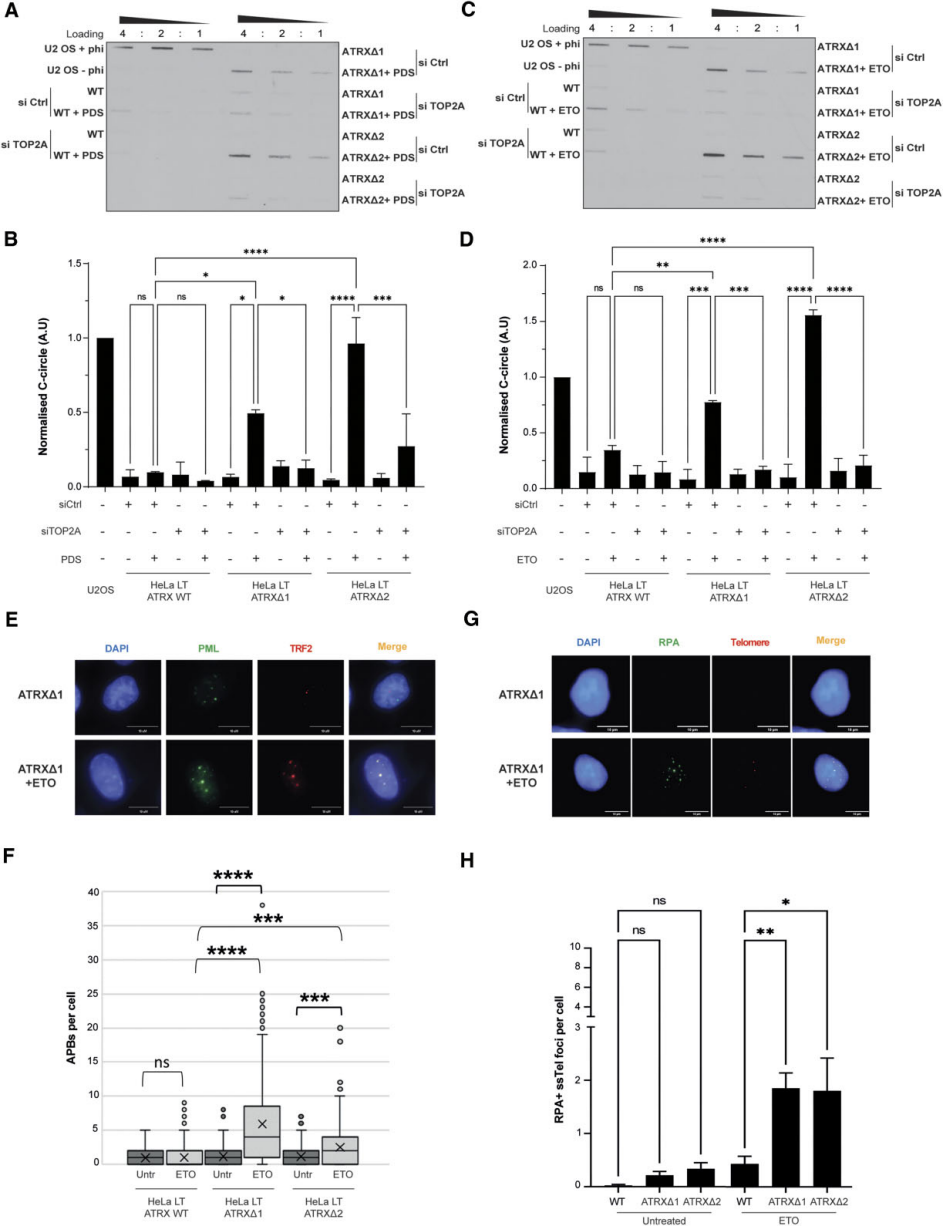
PDS and ETO lead to ALT induction in combination with ATRX loss through TOP2A trapping. (**A**) Representative image of C-circle blot of cells treated with 5 μM PDS for 48 h with and without siTOP2A knockdown. (**B**) Quantification of (A), two biological replicates run in triplicate. * *P* < 0.05, ** *P* < 0.01, *** *P* < 0.001, **** *P* < 0.0001, one-way ANOVA with Welch correction. (**C**) Representative image of C-circle blot of cells treated with 0.5 μM ETO for 48 h with and without siTOP2A knockdown. (**D**) Quantification of (C), two biological replicates run in triplicate. *** *P* < 0.0001, **** *P* < 0.0001, one-way ANOVA with Welch correction. (**E**) Representative immunofluorescence images of APB induction in HeLa LT ATRX knockout clones upon 48h treatment with 0.5 μM ETO. (**F**) Quantification of (E), >200 nuclei analysed across three biological replicates, *** *P* < 0.001, **** *P* < 0.0001, Kruskall–Wallis test. (**G**) Representative RPA ssTel immunoFISH images in HeLa LT ATRX knockout cells treated with 0.5 μM ETO for 48 h. (**H**) Quantification of RPA ssTel foci, >150 nuclei analysed across three biological replicates. * *P* < 0.05, ** *P* < 0.01, **** *P* < 0.0001, one-way ANOVA with Welch correction.

The rapid approach to DNA adduct recovery (RADAR) assay (46) was performed and this confirmed that PDS treatment led to a significant increase in trapped TOP2A levels on the DNA and—to a lesser extent—TOP1 (Supplementary Figures S6A and B). TOP2A was depleted using siRNA in the HeLa LT clones (Supplementary Figure S6C), and this abrogated the level of C-circles and APBs observed upon PDS treatment in ATRX null cells (Figures 2A, B and 1D), suggesting that PDS-initiated induction of ALT was indeed dependent on TOP2A. To confirm that this result was not specific to PDS, a second G4-stabiliser (CX-5461) was used; this also induced ALT hallmarks (Supplementary Figures S6D–F) and caused increased levels of trapped TOP2A (Supplementary Figure S6G), as previously reported (55,56).

To explore this pathway further, we next treated the cells with etoposide (ETO), a canonical TOP2 poison which directly stabilises the TOP2 cleavage complex (TOP2cc) on DNA. The increased trapping of TOP2A was confirmed by RADAR assay (Supplementary Figures S6A and B). Strikingly, treatment of the ATRX knockout clones with ETO elicited an increase in C-circles, APBs and RPA-ssTel foci to levels comparable to U2OS cells, whereas no notable increase was observed in the ATRX wildtype cells (Figures 2C–H). Knockdown of TOP2A decreased the effect of ETO, preventing the robust induction of ALT markers, therefore reinforcing the requirement for TOP2A trapping, as opposed to loss of activity, in triggering the ALT pathway upon loss of ATRX (Figures 2C, D).

### Trapping of other proteins induces ALT in ATRX-deficient cells

DNA protein covalent complexes (DPCCs) are formed as transient intermediates in a variety of protein-DNA interactions and more than 20 different proteins have been reported to form DPCCs (46). As such, we next considered whether the induction of ALT in ATRX-null cells was restricted to trapping of TOP2A, or whether the enforced trapping of other DNA interacting proteins could elicit the same effect.

**Figure 3.**
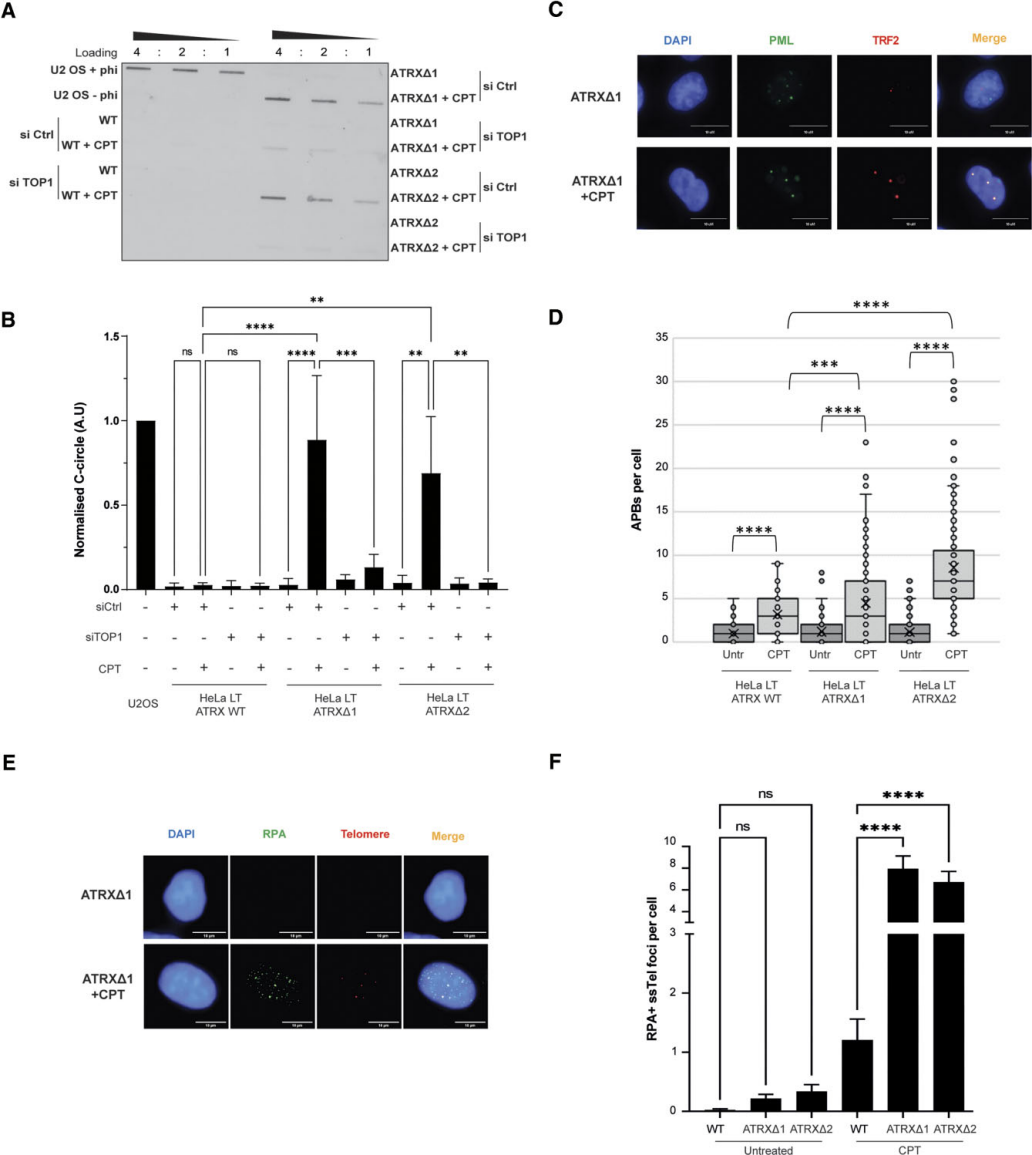
CPT leads to ALT induction in combination with ATRX loss through TOP1 trapping. (**A**) Representative image of C-circle blot of cells treated with 50 nM CPT for 48 h with and without siTOP1 knockdown. (**B**) Quantification of (A), three biological replicates run in triplicate. ** *P* < 0.01, *** *P* < 0.001, **** *P* < 0.0001, one-way ANOVA with Welch correction. (**C**) Representative immunofluorescence images of APB induction in HeLa LT ATRX knockout clones upon 48h treatment with 50 nM CPT. (**D**) Quantification of (C), >200 nuclei analysed across three biological replicates. *** *P*< 0.001, **** *P*< 0.0001, Kruskall–Wallis test. (**E**) Representative RPA ssTel immunoFISH images in HeLa LT ATRX knockout cells treated with 50 nM CPT for 48 h. (**F**) Quantification of (E), >150 nuclei analysed across 3 biological replicates. **** *P* < 0.0001, one-way ANOVA with Welch correction.

Topoisomerase I (TOP1) can be trapped onto DNA through treatment with TOP1 poisons such as camptothecin (CPT) and this effect was confirmed in our system (Supplementary Figure S6A and B). Treatment with CPT led to a strong induction of C-circles, APBs and RPA ssTel foci in ATRX-deficient cells. (Figures 3A–F). It was noted that, in the case of APBs, there was also a significant increase in ATRX wildtype cells treated with CPT, however the number of APBs were still significantly greater in the two ATRX knockout clones (Figure 3D). The same effect was noted in DAXX knockout clones (Supplementary Figures S7A–C). Induction of ALT was not observed when cells were treated with concurrent knockdown of TOP1 protein and CPT, consistent with the observed effect being due to trapping of TOP1 rather than loss of catalytic activity (Figures 3A, B and Supplementary Figures S8A and B).

PARP1, a central player in the DNA damage response, is also known to accumulate on chromatin when cells are treated with a variety of PARP inhibitors (PARPi). It should be noted, however, that rather than the covalent complexes formed by TOP1 (Top1cc) and TOP2A (Top2cc), the trapping of PARP1 on DNA is non-covalent in nature. It was recently demonstrated that the persistent accumulation of PARP1 foci upon treatment with PARPi was due to rapid and continuous recruitment and exchange of PARP1 at DNA lesions (57,58).

Notably, the potency of different inhibitors to trap PARP1 varies markedly, with allosteric differences in trapping behaviours (Supplementary Figure S9A). PJ34 is a catalytic inhibitor of PARP1 activity that has been reported to have no trapping action (59). Conversely, talazoparib is a PARPi which has consistently demonstrated extremely strong trapping potency (59–62). It has previously been reported that niraparib strongly traps PARP1, olaparib exhibits an intermediate level and veliparib exhibits no trapping potency, a pattern which does not correlate with the catalytic inhibitory properties for each drug (61–63). This dogma has, however, been challenged recently, with one group demonstrating that although olaparib does display trapping tendencies, niraparib might belong to the non-trapping class of agents (58). We considered it essential, therefore, to confirm the trapping potency of these agents in our cellular system.

**Figure 4.**
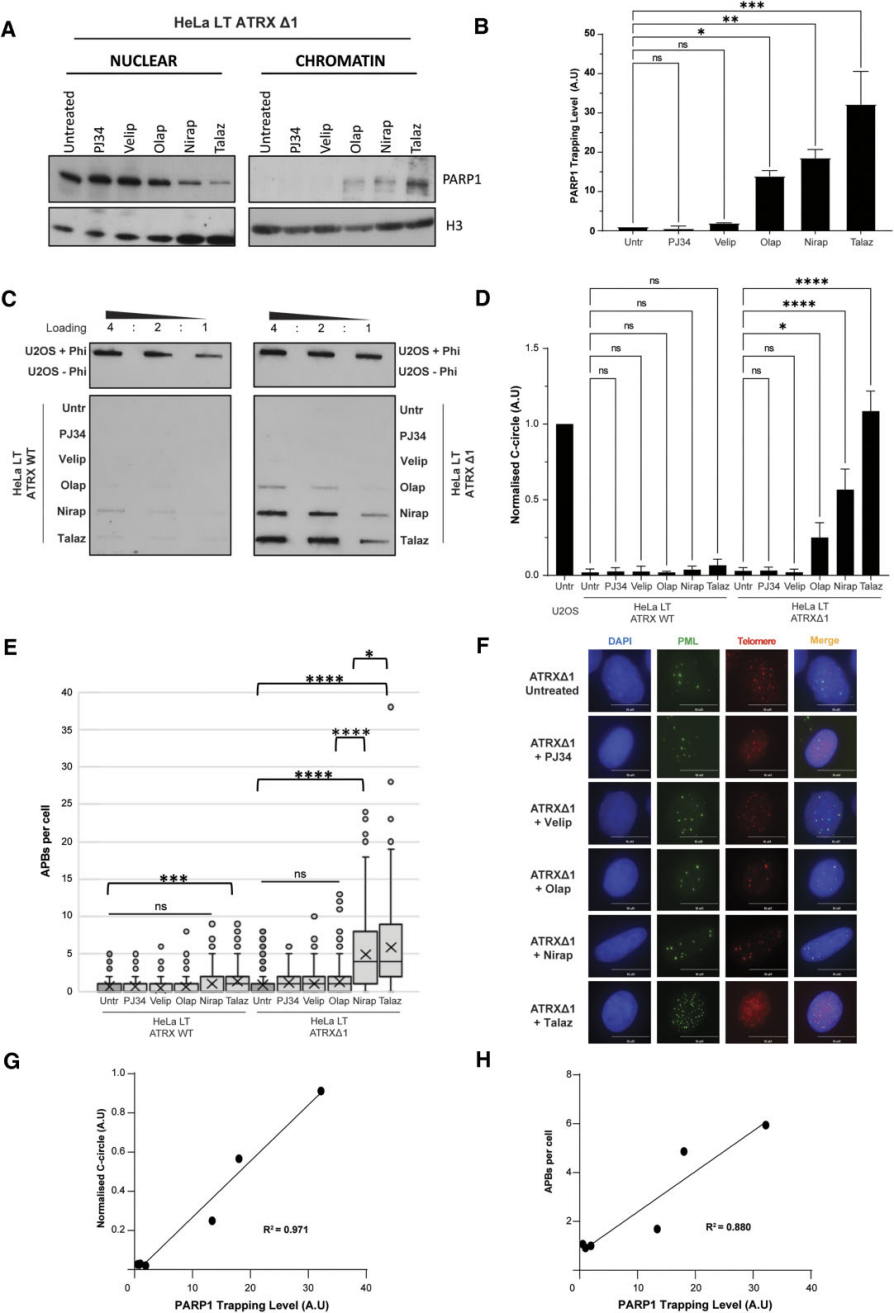
Trapping PARPi leads to ALT induction in combination with ATRX loss. (**A**) Representative immunoblot showing nuclear and chromatin bound fractions of HeLa LT ATRXΔ1 cells treated with 5 μM of the indicated PARPi for 48 h. (**B**) Quantification of (A), two biological replicates. * *P*< 0.05, ** *P* < 0.01, *** *P* < 0.001, **** *P* < 0.0001, one-way ANOVA with Welch correction. (**C**) Representative image of C-circle blot in HeLa LT ATRX WT and ATRXΔ1 cells treated with a range of trapping and non-trapping PARPi. (**D**) Quantification of (C), three biological replicates run in triplicate. * *P* < 0.05, **** *P* < 0.0001, one-way ANOVA with Welch correction. (**E**) Representative immunoFISH images of APB induction in HeLa LT ATRX knockout clones upon 48h treatment with various PARPi. (**F**) Quantification of (E), >200 nuclei analysed across three biological replicates, * *P* < 0.05, *** *P* < 0.001, **** *P* < 0.0001, Kruskall–Wallis test. (**G**) Linear regression analysis between PARP1 trapping level and C-circle level with the various PARPi drugs, *R*^2^ = 0.971, *P* = 0.0003. (**H**) Linear regression analysis between PARP1 trapping level and APBs per cell with the various PARPi drugs, *R*^2^ = 0.880, *P* = 0.0005

Nuclear and chromatin protein fractions were extracted from untreated cells and those treated with a PARPi panel. This demonstrated that PJ34 and veliparib did not induce trapping of PARP1, whilst olaparib induced a moderate amount of trapping (Figures 4A, B). Niraparib and talazoparib were both found to strongly trap PARP1 on chromatin, with talazoparib having even greater action than niraparib (Figures 4A, B). Importantly, all the drugs reduced PARylation levels to a similar degree (Supplementary Figure S9B). We took advantage of these properties to further test our hypothesis that it is the trapping of proteins on DNA that drives the ALT pathway. Treatment of ATRX wildtype cells with PARP1-inhibitors failed to induce C-circles (Figures 4C, D). Strikingly, however, treatment of both ATRX knockout clones with trapping PARPi led to an induction of C-circles (Figures 4C, D and Supplementary Figures S9C and S9D). Veliparib and PJ34 (non-trapping PARPi) failed to elicit any detectable C-circles, whilst olaparib, niraparib and talazoparib elicited a stepwise increase in C-circles (Figures 4C, D and Supplementary Figures S9C and S9D). There was a striking correlation between trapping potency and level of induced C-circles (*R*^2^ = 0.971, *P* = 0.0003) (Figure 4G). The same pattern was observed upon APB analysis (Figures 4E, F and Supplementary Figure S9E), with a similarly striking correlation (*R*^2^ = 0.880, *P* = 0.0005) (Figure 4H). As seen with CPT, there was a significant increase in APB levels in ATRX wildtype cells treated with the strongest trapper, talazoparib, however once again the effect in the ATRX knockout clones was significantly greater (Figure 4E). As seen with other agents, induction of ALT was not observed when cells were treated with concurrent knockdown of PARP1 protein and PARP trapping drugs, again confirming the mechanism of action as protein trapping, rather than loss of protein catalytic activity (Supplementary Figures S9F–H).

**Figure 5.**
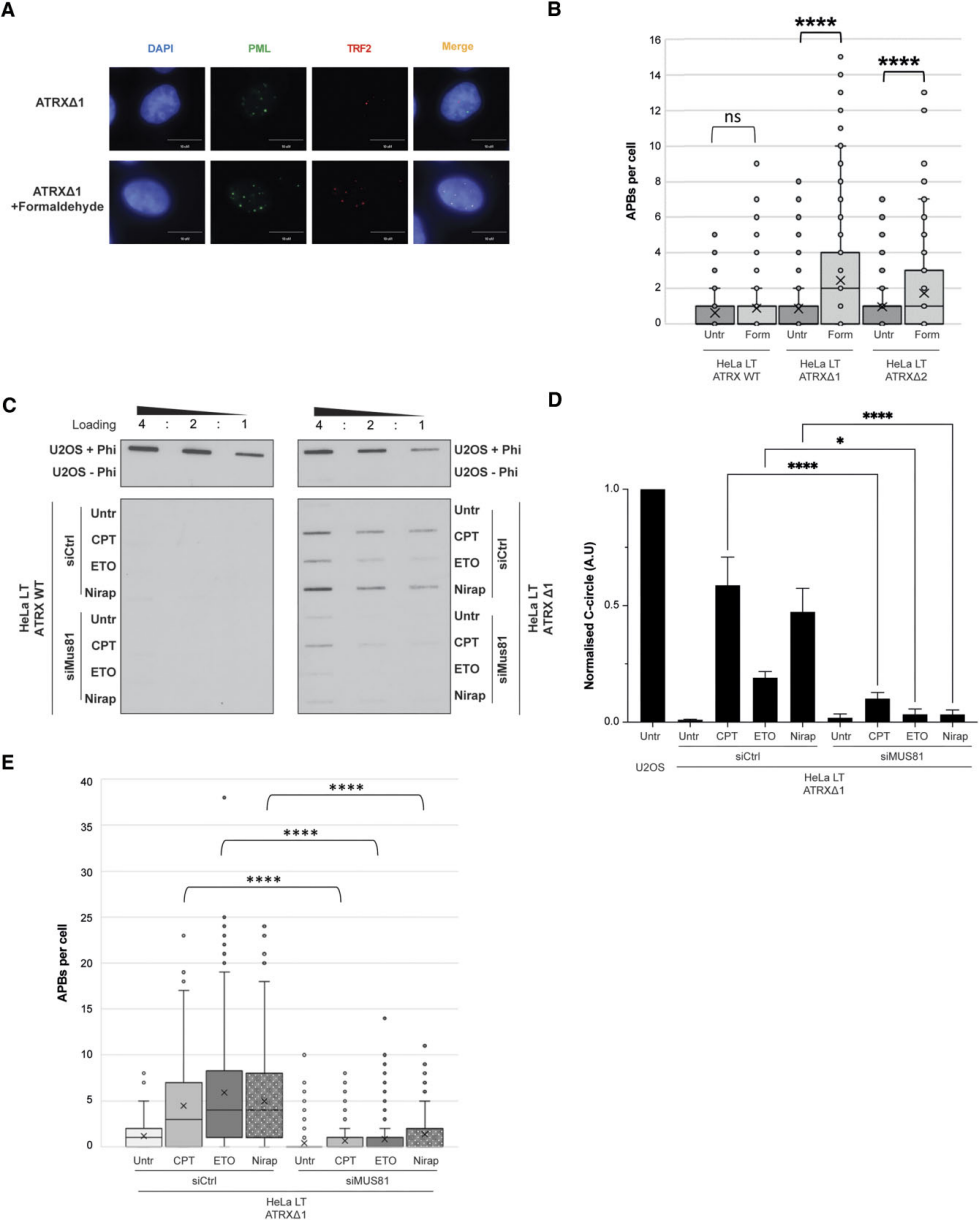
Formaldehyde crosslinking leads to ALT induction in combination with ATRX loss and induction of ALT is dependent on MUS81. (**A**) Representative immunofluorescence images of APB induction in HeLa LT ATRX knockout clones upon 1 h treatment with 500 μM formaldehyde and 48 h recovery time. (**B**) Quantification of (A), >200 nuclei analysed across three biological replicates, **** *P* < 0.0001. (**C**) Representative C-circle blot showing induction of C-circles upon ATRX loss and CPT/ETO/Niraparib treatment is dependent on MUS81. (**D**) Quantification of (C), three biological replicates run in triplicate. * *P* < 0.05, **** *P* < 0.0001. (**E**) Quantification of APB induction in HeLa LT ATRX knockout clones upon 48 h treatment with CPT/ETO/Niraparib in combination with siCtrl and siMUS81, >200 nuclei analysed across three biological replicates.

As the effect of protein trapping did not appear to be specific to any one protein, but rather a general effect of DPCC formation or protein trapping, we next investigated the effect of formaldehyde. Formaldehyde is a potent crosslinking agent, which forms covalent bonds between proteins and DNA. Treatment of the cells with formaldehyde had no effect on APBs in ATRX wildtype HeLa LT cells but caused a striking increase in ATRX-deplete clones, although curiously no C-circles could be amplified (Figures 5A, B and Supplementary Figure S10A). A possible explanation for this discrepancy is that formaldehyde is causing crosslinking of the GC-rich C-circle DNA, thereby prohibiting rolling circle amplification.

Taken together, this data suggested that it is the trapping of proteins on DNA that drives ALT in the absence of ATRX and not replicative stress *per se*. To further test this hypothesis, we treated the ATRX deficient cells with the DNA polymerase inhibitor aphidicolin (APH) and hydroxyurea (HU), at both low and high doses. APH has previously been shown to generate replicative stress at common fragile sites, including telomeres (8), but does not generate DPCCs, and hydroxyurea (HU) causes replicative stress through depletion of nucleosides. APH and HU treatments failed to generate a significant increase in C-circles or APBs in any HeLa LT cell clones, including the ATRX knockout clones—even when treated at high doses (Supplementary Figures S10B–F). Taken together, we conclude that trapping of proteins on DNA, in combination with ATRX loss, can induce the ALT pathway.

### Induction of ALT by trapped proteins is dependent on MUS81-endonuclease activity

Trapping of proteins on DNA leads to excessive replicative stress, because when a trapped protein is encountered by the replication fork, fork stalling and reversal will occur unless the trapping is resolved. We saw an increase in γH2AX levels in our ATRX knockout cells treated with trapping agents (Supplementary Figure S11A and S11B), indicative of replication fork collapse and the generation of DSBs. MUS81 endonuclease has been shown to reverse excessive DNA supercoiling resulting from protein trapping (64). Further, MUS81 localises to APBs in ALT cells, and depletion of MUS81 results in reduction of ALT specific-telomere recombination (65). During S-phase, the MUS81–EME2 complex is responsible for the restart of stalled replication forks and telomere recombination (66). It was considered, therefore, whether the observed induction of ALT-markers on trapping of proteins was dependent on MUS81 activity. Cells were treated with siRNA to MUS81 to knockdown expression (Supplementary Figure S11C). In contrast to drug treatment alone, when ATRX-deficient cells were co-treated with a protein trapping drug (ETO, CPT, niraparib or PDS) and siMUS81, there was minimal induction of markers of ALT (Figures 5C–E and Supplementary Figures S11D-F).

RPA stabilisation of single-stranded DNA (ssDNA) intermediates arising from a resected DSB, has previously been shown to be critical for BIR in yeast (67). Consistent with this, following treatment of cells with PDS, there was recruitment of RPA to telomeres in ATRX-deficient (but not ATRX-wildtype) cells (Supplementary Figures S11D and E). However, on co-treatment with PDS and siMUS81, there was a significant reduction in RPA recruitment to the telomeres, indicating that in the absence of MUS81, such ssDNA intermediates were not generated (Supplementary Figures S11D and E). We conclude, therefore, that the induction of ALT in the context of ATRX-loss and presence of DPCCs is dependent on MUS81 endonuclease activity and generation of DSBs by MUS81. Given the well-described role of MUS81 in resection of stalled replication forks (64–66), we believe that it is highly likely the observed DSBs arise from collapse of stalled replication forks.

### ATRX-deficient cells have higher levels of trapped protein on DNA

Finally, we investigated whether ALT+ cell lines have increased levels of trapped proteins. Previous proteomic work suggested that TOP1, TOP2A and PARP1 can all be detected at telomeres of the ALT+ U2OS cell line, potentially indicative of protein trapping at telomeres (Supplementary Figure S12A) (21). Re-analysis of this BioID data indicated that, in U2OS cells, there is a particular excess of TOP2A and TOP2B at telomeres, as compared to HeLa LT (21) (Supplementary Figure S12B). This might indicate that in this cell line, the predominate telomeric trapped protein is TOP2A/B, however, it is difficult to draw firm conclusions as the comparator is a non-isogenic line.

**Figure 6.**
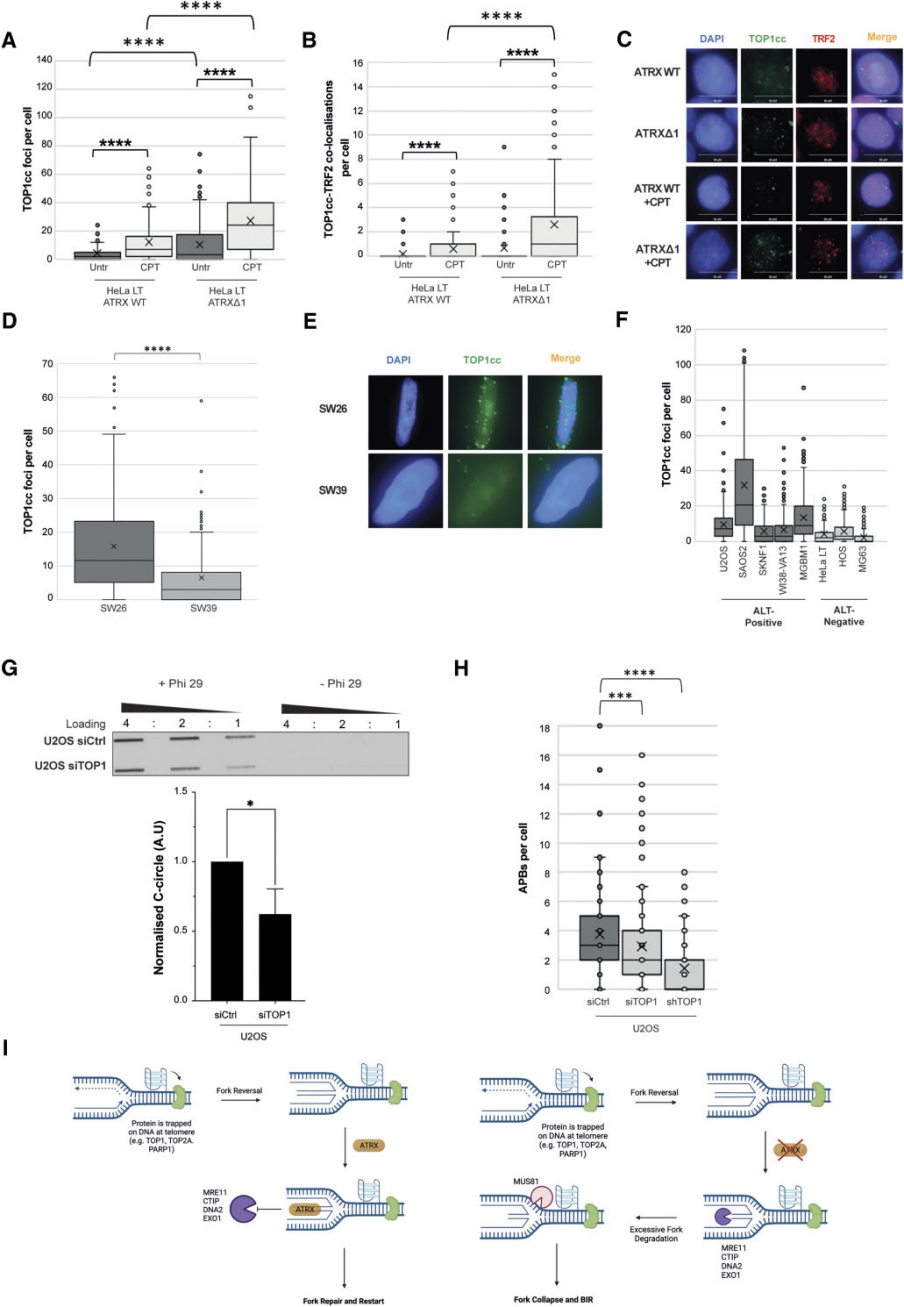
ALT cells have more trapped TOP1. (**A**) Quantification of immunofluorescence analysis showing increased number of Top1cc foci in HeLa cells upon CPT treatment, analysed across three biological replicates. **** *P* < 0.0001. (**B**) Quantification of immunofluorescence analysis showing increased co-localisation of TOP1cc and TRF2 in HeLa cells upon CPT treatment, analysed across 3 biological replicates. **** *P* < 0.0001. (**C**) Representative immunofluorescence images of (B) showing TOP1cc and TRF2 co-localisation. (**D**) Quantification of immunofluorescence analysis showing increased levels of TOP1cc foci in the ALT+ cell line SW26 versus the ALT– cell line, >200 nuclei analysed across three biological replicates. **** *P*< 0.0001. (**E**) Representative immunofluorescence images of (D) showing TOP1cc foci. (**F**) Quantification of immunofluorescence analysis showing generally increased levels of TOP1cc foci in ALT+ cell lines versus ALT–, analysed across two biological replicates. (**G**) Representative C-circle assay and quantification of U2OS cells treated with siCtrl and siTOP1, three biological replicates run in triplicate, * *P* < 0.05, unpaired *t*-test. (**H**) Quantification of APBs in U2OS cells treated with siCtrl, siTOP1 and shTOP1, >200 cell analysed across three biological replicates. *** *P*< 0.001, **** *P*< 0.0001, Kruskall–Wallis test. (**I**) Proposed model of ALT induction. Proteins, such as TOP1, TOP2A and PARP1 become trapped at telomeres, potentially by non-canonical secondary structures such as G4s and R-loops, leading to fork stalling. In the presence of ATRX, forks are protected and can be repaired and restarted (top panel). In the absence of ATRX (bottom panel), forks are no longer protected, leading to excessive nucleolytic degradation, cleavage by the MUS81 structure-specific endonuclease and fork collapse and subsequent BIR.

We next took advantage of a monoclonal antibody that specifically recognises TOP1cc but not free TOP1 or DNA (68). Consistent with a specificity of the antibody for TOP1cc, immunofluorescence analysis showed a significant increase in the levels of both total and telomeric TOP1cc in both ATRX wildtype and deplete HeLa LT cells upon treatment with CPT (Figures 6A–C). We note that levels of TOP1cc were generally higher in the ATRX deplete cells, and this increase was exacerbated upon CPT treatment (Figures 6A–C). Importantly, when comparing the ALT + cell line SW26 with the isogenic telomerase positive, ALT– cell line SW39 (69), we found that the ALT+ cell line exhibited 2.4-times higher levels of trapped TOP1 protein as compared to its ALT- isogenic counterpart; the result was strongly significant (*t* = 6.22, *P* < 0.0001) (Figures 6D, E). The level of TOP1cc in a panel of natural ALT+ cell lines was also assessed; this demonstrated that ALT+ cells generally had higher genome-wide levels of trapped TOP1 protein in comparison to ALT– cells (Figure 6F).

It has previously been shown that knockdown of the histone chaperones ASF1a and ASF1b in HeLa LT cells leads to induction of the ALT pathway (21). We knocked down ASF1 in our HeLa LT cells (Supplementary Figure S13A) and confirmed this led to induction of the ALT pathway (Supplementary Figure S13B). Strikingly, this induction of ALT correlated with a significant increase in TOP1cc levels (Supplementary Figures S13C and D), indicating that trapping of proteins on DNA is strongly linked to ALT-induction in these cells.

Next, the effect of protein trapping in the canonical ALT+ osteosarcoma cell line, U2OS, was explored. Crucially, depletion of TOP1 through both siRNA and shRNA (Supplementary Figure S14A) led to a significant reduction in both APBs and C-circles in U2OS (Figure 6G and H). We also tested another ALT+ cell line, MGBM1, however in this case found no effect on C-circle levels and only a minor effect on APBs (Supplementary Figures S14B and S14C). This suggested that in natural ALT+ cancer cells, formation of TOP1cc is not the only natural cause of ALT activity, consistent with our earlier observations that trapping of various proteins can induce the phenotype.

To explore this further we knocked down TOP2A (Supplementary Figure S14D) and found there was a decrease in C-circle levels in both MGBM1 and U2OS cell lines (Supplementary Figures S14E and S14F). There was a modest but statistically significant decrease in APBs observed in MGBM1, but no observed difference in U2OS cells (Supplementary Figure S14G). Finally, knockdown of PARP1 using shRNA in U2OS (Supplementary Figure S14H) led to a significant decrease in both C-circles and APBs (Supplementary Figures S14I-K). Knockdown of PARP1 was additionally attempted in MGBM1, but the cells were not viable.

## DISCUSSION

DNA is always associated with various proteins as part of normal genomic processes—however these frequent, close interactions carry the risk of formation of either DNA-protein covalent cross-links (DPCCs) or non-covalent trapping of proteins on DNA. Many enzymes—such as topoisomerases, polymerases, methyltransferases, glycosylases and poly(ADP-ribose) polymerases—form reversible covalent intermediates with DNA during catalysis, and these intermediates might accidentally form DPCCs (70). DPCCs are ubiquitous in the genome but are bulky and can, therefore, interfere with vital processes such as DNA replication, transcription, chromatin remodelling, and DNA topology manipulation (71–76). DPCCs can be generated by a variety of endogenous or environmental sources, including chemotherapeutical drugs, ionizing or UV radiation and oxidative stress, as well as erroneous enzymatic activity of normal DNA-interacting proteins. Enhancement of protein trapping has become an important mechanism of action for chemotherapeutic agents, such as etoposide, camptothecin-derivatives (such as irinotecan), and niraparib. It has been known for some time that ALT cancers are particularly sensitive to these agents, but the link between the ALT pathway and DPCC formation has not previously been investigated.

Here, it was demonstrated that several chemotherapeutic agents were capable of inducing ALT in HeLa LT cells which lacked ATRX, but not in ATRX wildtype clones. The agents which were able to induce ALT markers in the ATRX-deplete cells were pyridostatin and CX-5461 (G4 stabilising agents that trap TOP2A), camptothecin (TOP1 poison), etoposide (TOP2 poison), and niraparib and talazoparib (PARP1 trappers). The same effect was observed when the ATRX-deplete clones were treated with formaldehyde, an agent which induces widespread cross-links between protein and DNA. Crucially, olaparib (a less potent PARP1 trapping agent) did not induce markers of ALT to the same extent as niraparib or talazoparib, and PJ34 and veliparib (non-trapping PARP1 inhibitors) did not induce any markers of the ALT phenotype. Further, treatment with aphidicolin and hydroxyurea failed to elicit an increase in cardinal ALT markers in ATRX-deplete cells. These data strongly support the concept that it is not replicative stress *per se* driving ALT in ATRX-deplete cells, but replicative stress specifically caused by trapping of proteins.

It was considered likely, therefore, that trapping of proteins—including TOP1, TOP2 and PARP1—on DNA was a crucial step in initiation of ALT processes—and so it was conjectured that depletion of the implicated proteins should not have the same effect as poisons. It was demonstrated that depletion of TOP1 and PARP1 in two naturally occurring ALT lines (U2OS and MGBM1) led to a significant decrease in APBs; the effect of depletion of TOP2 led to a small decrease in APBs in MGBM1 but not U2OS. The effect on C-circle levels was less clear cut, with a decrease observed on silencing of PARP1 and TOP2A, but not on TOP1. It is likely, then, that the effect is not specific to one protein, but rather a generic effect of protein trapping, the magnitude of the effect possibly being related to relative gene expression levels, temporospatial relations or even stochastic effects. Further proteomic investigations into these questions might reveal insights into ALT-cancer biology.

Next, we considered the role of MUS81-endonuclease, an enzyme which has been implicated in the repair of DNA in response to protein trapping, restart of stalled replication forks and telomere recombination in ALT-cells (64–66). As expected, in the absence of MUS81, protein-trapping drugs were unable to induce markers of ALT in ATRX-deficient cells. Further, ALT telomere synthesis has previously been demonstrated to be reliant on BLM and the BIR pathway; here, we demonstrated that the induction of ALT observed under conditions of protein trapping was, indeed, dependent on these pathways. This confirmed that in our artificial ATRX knockout cellular system, trapping of proteins was likely initiating ALT through the same pathway that naturally occurs in ALT + malignant cells: that is, MUS81- and BLM-dependent break-induced replication. Crucially, it has been demonstrated that MUS81 endonuclease has a role in reversal of excessive DNA supercoiling resulting from protein trapping, as well as cleavage of replication intermediates in G2, formed as a result of DNA protein cross-links, to facilitate BIR (64,77). Taken together, these findings provide a cogent model of ALT-biology, where trapped proteins lead to stalling of replication fork progression, with subsequent recruitment of MUS81 and fork resection, which provides the genetic substrate for BLM-mediated BIR, but only in the absence of ATRX/DAXX (Figure 6I).

An important consideration is whether protein trapping is one potential natural driving force behind ALT, or an artificial way that ALT can be induced in cellular systems using chemotherapeutic agents. Firstly, we showed that knockdown of ASF1 also elicited an increase in TOP1cc and concurrent induction of ALT; this supports the conclusion that accumulation of trapped proteins is intrinsic to the ALT process, rather than a coincidental event. Further, we showed that natural ALT + cell lines exhibit higher baseline levels of trapped TOP1cc relative to ALT- cells, including in isogenic paired ALT+/ALT- lines (SW26/SW39). It is unlikely, however, that a single trapped protein is responsible for this phenomenon. We observed that knockdown of TOP1, TOP2A, PARP1 in two ALT cell lines – U2OS and MGBM1 – had differing magnitudes of effect, indicating cell-specific differences. Further, it was previously demonstrated that TOP2cc were enriched at telomeres in ATRX-deficient cells (78). Future work to characterise the repertoire of trapped telomeric proteins across ALT cancer cells will be of great interest and help answer these questions.

Another important consideration concerns the role of ATRX in preventing fork collapse in the presence of protein trapping. ATRX has previously been implicated with multiple roles at the replication fork which could explain the data we present here, including roles in both the protection of stalled forks from nucleolytic degradation (33) and the restart of stalled replication forks (34,37). Of note, ATRX has been proposed to interact with factors known to modulate both fork restart and protection and has also been shown to interact and cooperate with FANCD2 to recruit CtIP to stalled replication forks and promote MRE11 dependent fork restart (37). ATRX itself has also been shown to interact with components of the MRN complex, raising the intriguing possibility that ATRX constitutes a component of a fork restart complex (20,34,37). Understanding how these interactions are regulated will likely give important insights as to how ATRX facilitates progressive DNA replication in the presence of trapped proteins on DNA.

Taken together, these data strongly suggest that trapping of protein on DNA is one potential key driving force in the natural aetiology of ALT, although why ALT cells should have higher levels of trapped proteins remains to be explored. Interestingly, we observed that knock out of *ATRX* in the HeLa LT cell line in itself elicited an increase in TOP1cc levels. This data is consistent with previous findings which has shown that ATRX loss is associated with increases in R-loops, including telomeric R-loops (29) which have been linked to increases in DNA supercoiling and protein trapping. However, given that these cells do not exhibit canonical markers of ALT prior to treatment with genotoxic agents, we can conclude that this small increased basal level of TOP1cc is not sufficient to trigger the ALT pathway in isolation. Within a tumour, however, we must consider the heterogenous cellular population due to clonal evolution, and the effect of tumour microenvironment. It is likely that the increased level of trapped protein in ATRX-deficient cells drives the initiation of ALT when in the context of other genetic, metabolic or cellular challenges that further increase the levels of DPCCs.

Of note, the human cancers in which ALT is most prominent are also those most likely to harbour mutations in *IDH1* (2). The *IDH1* R132H mutation leads to the accumulation of the oncometabolite 2-hydroxygluterate (2-HG) which in turn inhibits multiple enzymes related to cytosine and histone methylation, resulting in perturbations in the expression of multiple genes. Recent research has shown that *IDH1* R132H mediated loss of *XRCC1* expression is key to ALT induction in ATRX deplete cells (79). Of note a novel role has recently been identified for XRCC1 in the prevention of trapping of PARP1 on single strand break intermediates during base excision repair (80). As such, it is tempting to speculate that IDH1 mutant ALT cancers may exhibit high levels of trapped PARP1 through loss of XRCC1 protein activity.

The formation of TOP1cc can be facilitated by the interaction of TOP1 with oxidised bases such as 7,8-dihydro-8-oxoguanine (8-oxoG) (81), suggesting that the accumulation of oxidative stress and reactive oxygen species (ROS) in ALT cancers might serve as a pathway to trap TOP1. Consistent with this possibility, telomerase suppression in a mouse lymphoma model has been found to lead to development of ALT+ tumours and, of note, the resultant tumours characteristically exhibited mitochondrial dysfunction and increased levels of ROS (82). Each of these hypotheses will need to be experimentally explored in more detail to fully elucidate the mechanisms underlying the observed phenomenon.

Here, we propose a model whereby, in the absence of ATRX or DAXX, forks become unstable upon encountering a DPCC. Given that many proteins have been purported to have a role in fork stability this raises the important question as to why ATRX appears to be the dominant tumour suppressor for ALT cancers? We consider the likely explanation for this phenomenon is the reported role for ATRX in the maintenance of telomere sister chromatid cohesion, thereby allowing for out of register BIR and net changes in telomere length (22,83). As such, this dual functionality of ATRX means loss of ATRX is uniquely dangerous for the activation of ALT.

Finally, the translational and clinical implications of these findings are of high importance. TOP1/TOP2A and PARP poisons are already an important component of many treatment protocols for glioblastoma, sarcoma and other ALT + tumour types. However, understanding that protein trapping induces a hyper-ALT phenotype should inform more rational drug combination therapy—for example, combining a non-trapping PARPi (such as veliparib) with a trapping agent (such as irinotecan) could be non-additive at best, or antagonistic at worst. It should also inform combinations with non-trapping based drugs—for example, studies have demonstrated that *FANCM* loss leads to a hyper-ALT phenotype, and so we could predict that combination of these agents with trapping drugs will have a synergistic effect (10–12). It is also hoped that further insight into the basic biology of ALT cancer cells could lead to the design of more novel targeted chemotherapeutic agents and, eventually, to improved outcomes for individuals with ALT+ cancers.

## Supplementary Material

gkad150_Supplemental_File

## Data Availability

ImageJ scripts for z-projecting of Deltavision image files together with CellProfiler pipelines for foci quantification and co-localisation analysis of the projected images are available at https://github.com/CLYNESLAB/Image-Analysis and https://doi.org/10.5281/zenodo.7630004. All other data is contained within the manuscript and/or supplementary files.
