# Supplementary material for: Induction of the alternative lengthening of telomeres pathway by trapping of proteins on DNA
Source: Nucleic Acids Res. 2023 Mar 21;51(13):6509–27. doi: 10.1093/nar/gkad150 (PMC10359465; doi:10.1093/nar/gkad150)
Supplement: gkad150_Supplemental_File [file gkad150_supplemental_file.pdf]

FIGURE S1

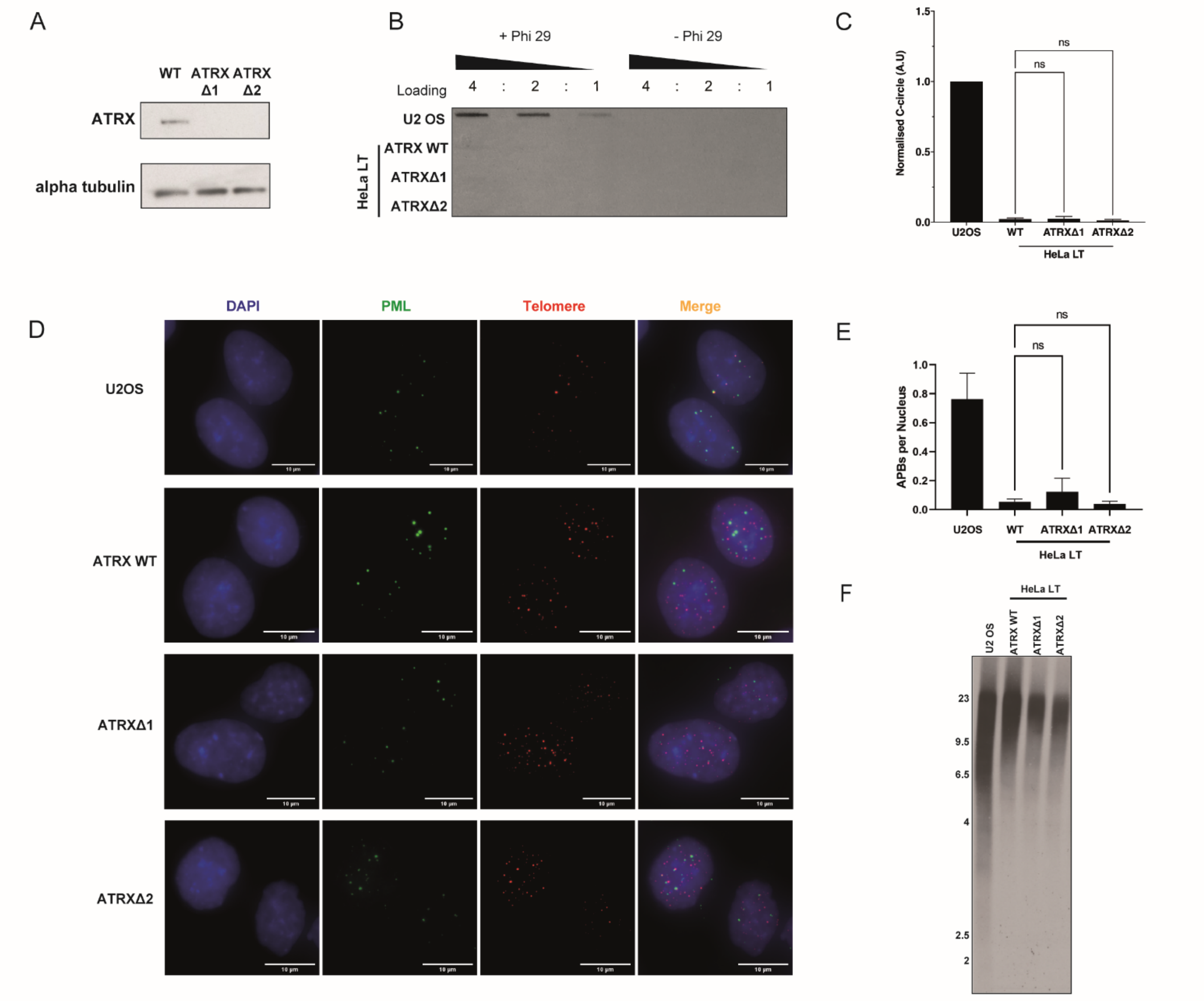

Supplementary Figure S1. Generation of HeLa LT ATRX knockout clones. A) Immunoblot confirming CRISPR-Cas9 mediated knockout of ATRX in two independent clones of HeLa LT. B) Representative C-circle blot showing that deletion of ATRX in the HeLa LT cell line is insufficient to trigger the accumulation of C-circles. C) Quantification of B, 3 biological replicates run in triplicate, one-way ANOVA with Welch correction. D) Representative immunoFISH images of APBs in the HeLa LT clones, showing no induction of APB formation upon ATRX loss. E) Quantification of D, > 200 nuclei analysed across 3 biological replicates, one-way ANOVA with Welch correction. F) Terminal Restriction Fragment (TRF) analysis of telomere length showing no overt changes in telomere length or heterogeneity upon ATRX deletion.

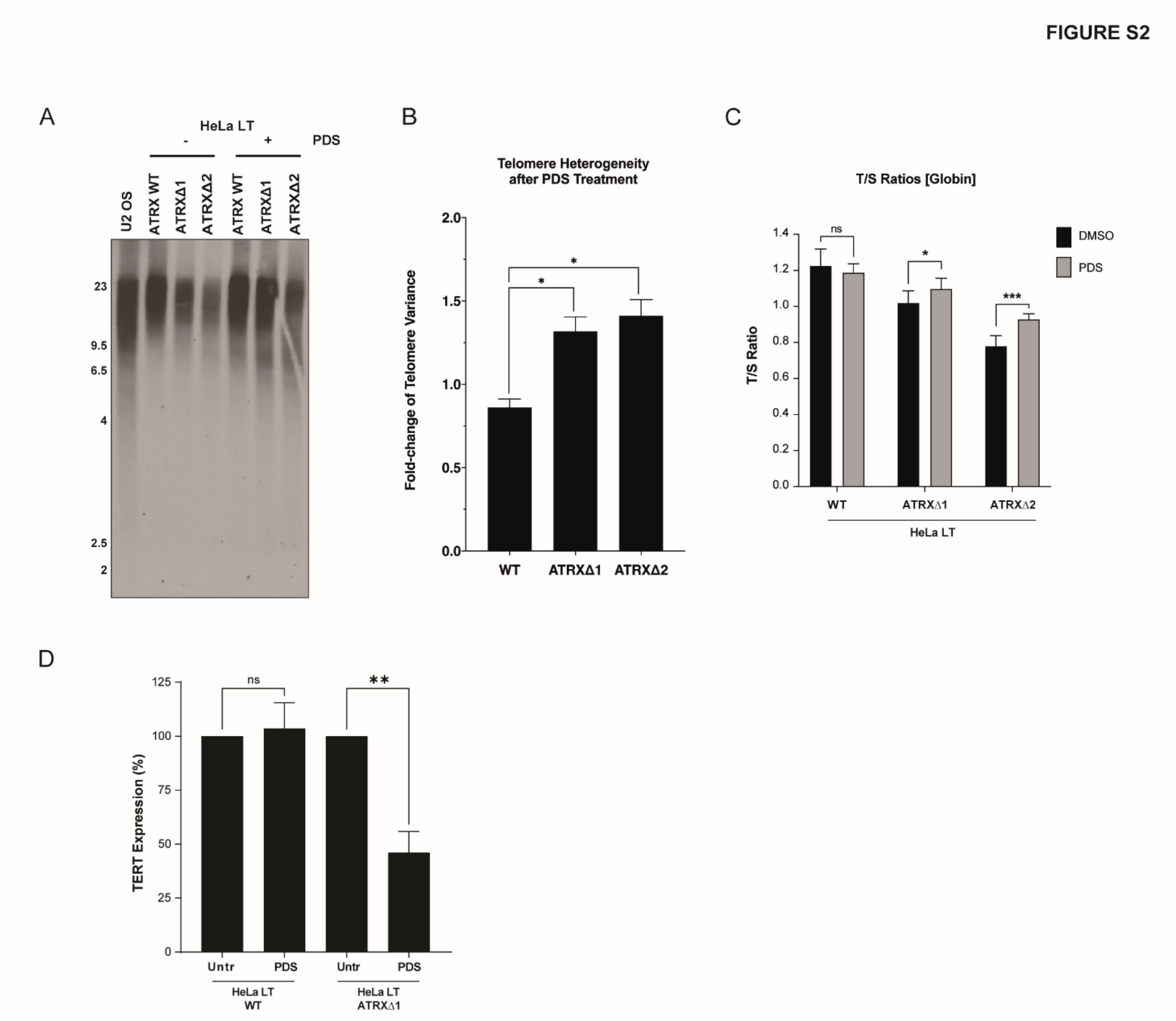

Supplementary Figure S2. Treatment with PDS in combination with ATRX loss triggers further ALT markers. A) TRF analysis showing treatment with PDS increased telomere length heterogeneity specifically upon depletion of ATRX. B) Telomere heterogeneity of blot in A was measured using TeloMetric software, 2 biological replicates. * $P < 0.05$, one-way ANOVA with Welch correction. C) Ratio of telomere repeats to the single copy gene beta-globin (T/S ratio) following the addition of PDS as assessed by mm-qPCR in HeLa LT ATRX wildtype or ATRX knockout clones, 6 biological replicates run in triplicate. * $P < 0.05$, *** $P < 0.0001$, one-way ANOVA with Welch correction. D) RT-qPCR analysis of TERT expression levels in the HeLa WT and ATRXΔ1 in the presence and absence of PDS. Expression levels were normalised to 7SK levels and the untreated samples by the ΔΔCT method. Results from two biological replicates run in triplicate. ** $P < 0.01$, one-way ANOVA with Welch correction.

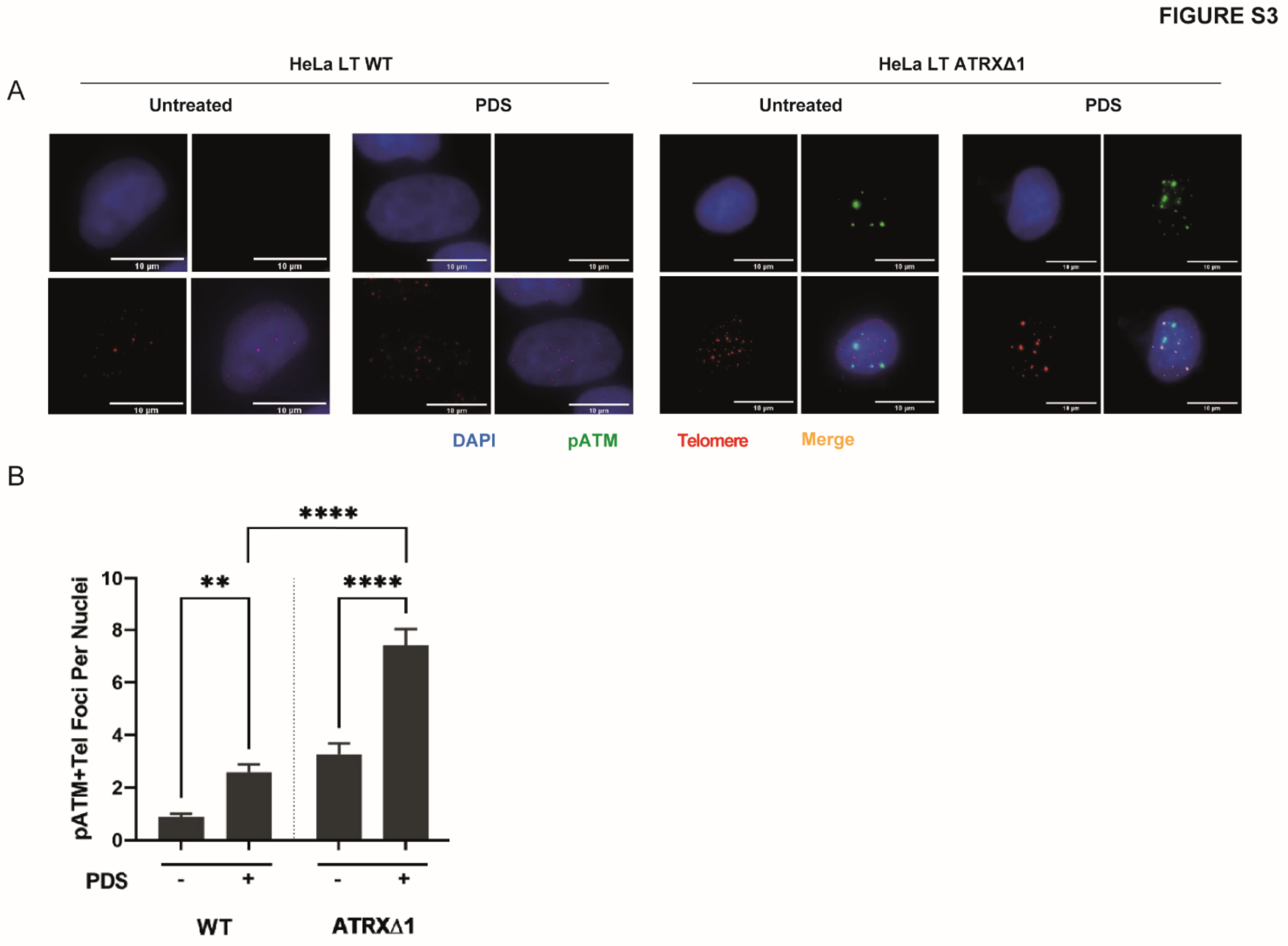

Supplementary Figure S3. Treatment with PDS in combination with ATRX leads to telomere dysfunction induced foci. A) Representative ImmunoFISH images showing co-localisation of pATMS1981 with telomeres. B) Quantification of A, > 200 nuclei analysed across 3 biological replicates. ** $P < 0.01$, **** $P < 0.0001$, one-way ANOVA with Welch correction.

FIGURE S4

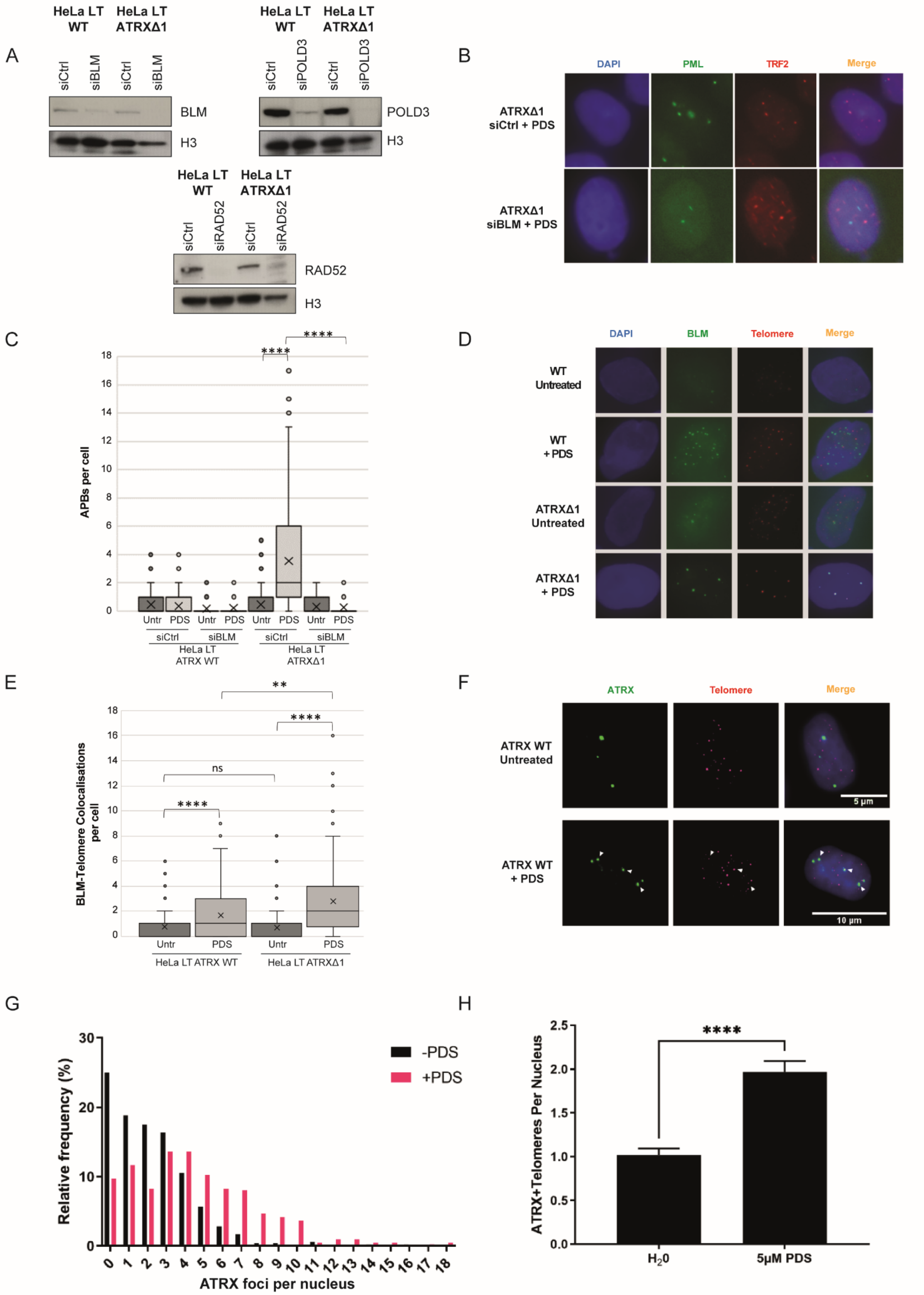
A
HeLa LT WT
HeLa LT ATRXΔ1
siCtrl
siBLM
siCtrl
siBLM
BLM
H3
siPOLD3
POLD3
siRAD52
RAD52
B
DAPI
PML
TRF2
Merge
ATRXΔ1 siCtrl + PDS
ATRXΔ1 siBLM + PDS
C
APBs per cell
Untr
PDS
HeLa LT ATRX WT
HeLa LT ATRXΔ1
D
BLM
Telomere
WT Untreated
WT + PDS
ATRXΔ1 Untreated
ATRXΔ1 + PDS
E
BLM-Telomere Colocalisations per cell
ns
F
ATRX
ATRX WT Untreated
ATRX WT + PDS
5 µm
10 µm
G
Relative frequency (%)
-PDS
+PDS
ATRX foci per nucleus
H
ATRX+Telomeres Per Nucleus
H20
5µM PDS

Supplementary Figure S4. Induction of ALT in ATRX knockout cells treated with PDS is dependent on the BIR pathway. A) Immunoblot confirming siRNA-mediated knockdown of BLM, POLD3 and RAD52 in ATRX in HeLa LT WT and HeLa LT ATRXΔ1 cell lines. B) Representative immunofluorescence images of APBs in HeLa LT ATRXΔ1 cells treated with PDS in an siCtrl and siBLM background. C) Quantification of B, > 200 nuclei analysed across 2 biological replicates. **** P < 0.0001, Kruskall-Wallis Test. D) Representative immunoFISH images of BLM-telomere co-localisations in HeLa LT WT and ATRXΔ1 cells treated with PDS. E) Quantification of B, > 200 nuclei analysed across 2 biological replicates. ** P < 0.01, **** P < 0.0001, Kruskall-Wallis Test. F) Representative immunoFISH images showing ATRX recruitment to telomeres following treatment with PDS. G) Quantification of ATRX foci per nucleus in the presence and absence of PDS treatment. H) Quantification of ATRX-telomere co-localisations in the presence and absence of PDS treatment, > 200 nuclei analysed across 3 biological replicates. **** P < 0.0001, unpaired t-test.

FIGURE S5

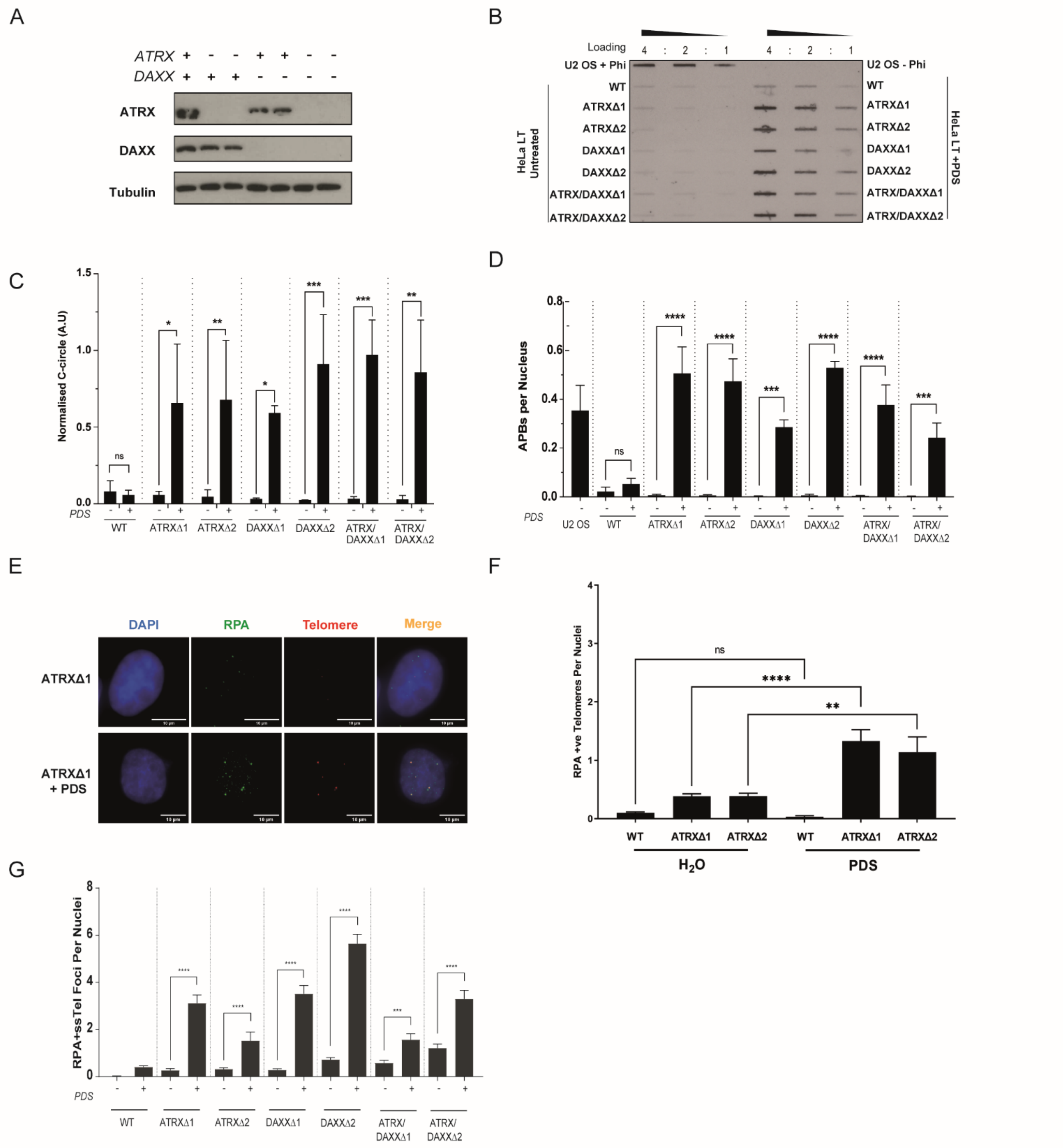
A
ATRX
DAXX
ATRX
DAXX
Tubulin
B
Loading
4 : 2 : 1
4 : 2 : 1
U2 OS + Phi
U2 OS - Phi
WT
ATRXΔ1
ATRXΔ2
DAXXΔ1
DAXXΔ2
ATRX/DAXXΔ1
ATRX/DAXXΔ2
HeLa LT Untreated
HeLa LT +PDS
C
Normalised C-circle (A.U)
PDS
WT
ATRXΔ1
ATRXΔ2
DAXXΔ1
DAXXΔ2
ATRX/ DAXXΔ1
ATRX/ DAXXΔ2
D
APBs per Nucleus
PDS
U2 OS
WT
ATRXΔ1
ATRXΔ2
DAXXΔ1
DAXXΔ2
ATRX/ DAXXΔ1
ATRX/ DAXXΔ2
E
DAPI
RPA
Telomere
Merge
ATRXΔ1
ATRXΔ1 + PDS
F
RPA +ve Telomeres Per Nuclei
ns
WT
ATRXΔ1
ATRXΔ2
WT
ATRXΔ1
ATRXΔ2
H2O
PDS
G
RPA+ssTel Foci Per Nuclei
PDS
WT
ATRXΔ1
ATRXΔ2
DAXXΔ1
DAXXΔ2
ATRX/ DAXXΔ1
ATRX/ DAXXΔ2

Supplementary Figure S5. ATRX and DAXX act epistatically in the suppression of PDS-induced ALT. A) Immunoblot of CRISPR-Cas9 mediated DAXX knockouts in HeLa LT ATRX WT and HeLa LT ATRXΔ1 cell lines. B) Representative C-circle blot in ATRX, DAXX and ATRX/DAXX knockout clones following treatment with PDS. C) Quantification of B, 3 biological replicates run in triplicate. * $P < 0.05$, ** $P < 0.01$, *** $P < 0.001$, one-way ANOVA with Welch correction. D) Quantification of APBs following PDS treatment in ATRX/DAXX knockout clones, > 200 nuclei analysed across 3 biological replicates. ** $P < 0.01$, *** $P < 0.001$, **** $P < 0.0001$, one-way ANOVA with Welch correction. E) Representative immunoFISH images of RPA ssTel foci in ATRX knockout clones following PDS treatment. F) Quantification of E, > 200 nuclei analysed across 3 biological replicates. ** $P < 0.01$, **** $P < 0.0001$, one-way ANOVA with Welch correction. G) Quantification of RPA ssTel foci in ATRX, DAXX and ATRX/DAXX knockout clones following PDS treatment, > 200 nuclei analysed across 3 biological replicates. *** $P < 0.001$, **** $P < 0.0001$.

FIGURE S6

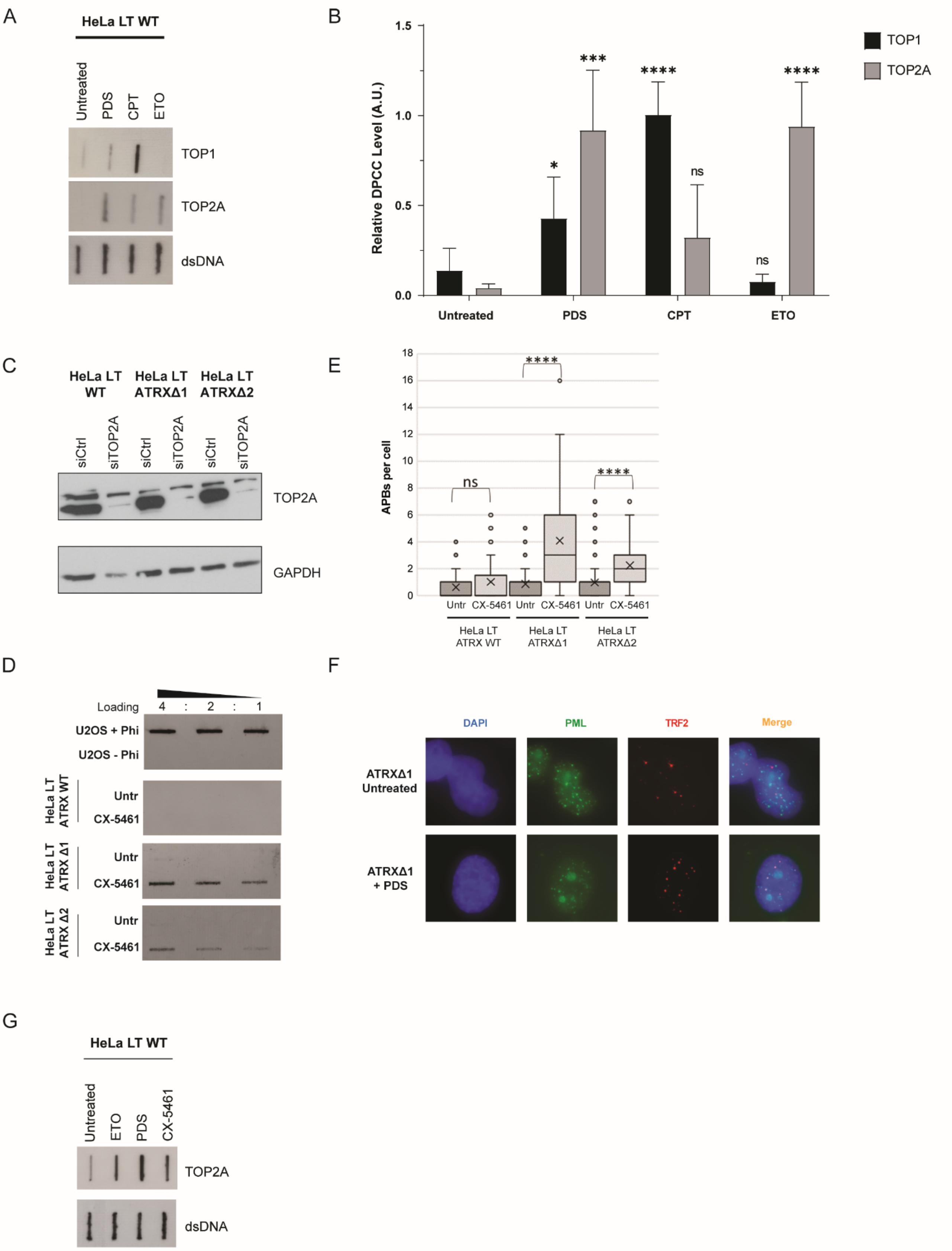
A
HeLa LT WT
Untreated
PDS
CPT
ETO
TOP1
TOP2A
dsDNA
B
Relative DPCC Level (A.U.)
1.5
1.0
0.5
0.0
TOP1
TOP2A
Untreated
PDS
CPT
ETO
ns
C
HeLa LT WT
HeLa LT ATRXΔ1
HeLa LT ATRXΔ2
siCtrl
siTOP2A
TOP2A
GAPDH
E
APBs per cell
Untr
CX-5461
HeLa LT ATRX WT
HeLa LT ATRXΔ1
HeLa LT ATRXΔ2
D
Loading
4 : 2 : 1
U2OS + Phi
U2OS - Phi
HeLa LT ATRX WT
HeLa LT ATRX Δ1
HeLa LT ATRX Δ2
Untr
CX-5461
F
DAPI
PML
TRF2
Merge
ATRXΔ1 Untreated
ATRXΔ1 + PDS
G
HeLa LT WT
Untreated
ETO
PDS
CX-5461
TOP2A
dsDNA

Supplementary Figure S6. PDS and CX-5461 lead to ALT induction in combination with ATRX loss through TOP2A trapping. A) Representative image of the RADAR assay, measuring levels of TOP1cc and TOP2Acc, following 1 hour treatment of HeLa LT WT cells with PDS, CPT and ETO. B) Quantification of A, 5 biological replicates. * $P < 0.05$, *** $P < 0.001$, **** $P < 0.0001$, one-way ANOVA with Welch correction. Significance levels refers to untreated equivalent. C) Immunoblot confirming siRNA-mediated knockdown of TOP2A in HeLa LT WT, ATRXΔ1 and ATRXΔ2 cell lines. D) C-circle assay blot showing induction of C-circles in ATRX knockout cells treated with 1 µM of CX-5461 for 48h. E) Quantification of APBs following treatment of ATRX knockout cells with 1 µM of CX-5461 for 48h, > 200 nuclei analysed across 2 biological replicates. **** $P < 0.0001$. F) APBs representative images. G) RADAR assay blot showing increased TOP2A trapping on DNA following 1h treatment of ETO, PDS and CX-5461.

FIGURE S7

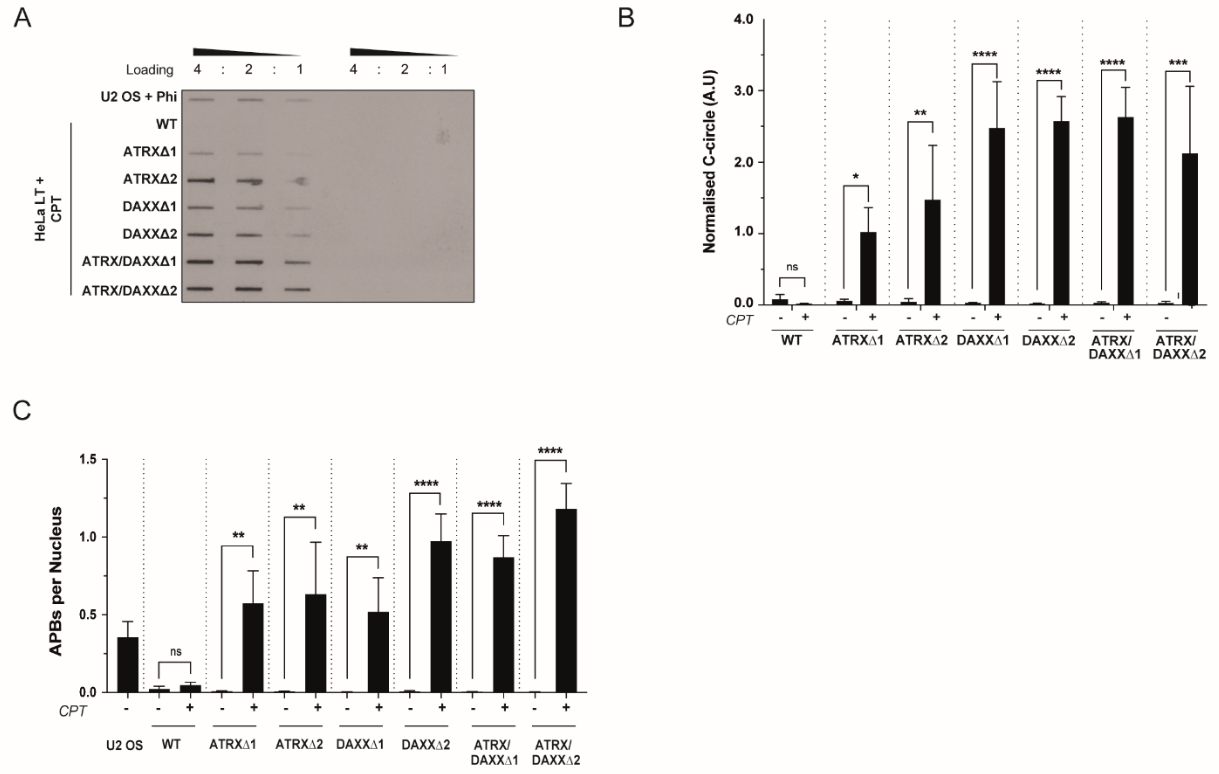

Supplementary Figure S7. ATRX and DAXX act epistatically in the suppression of CPT-induced ALT. A) Representative C-circle blot in ATRX, DAXX and ATRX/DAXX knockout clones following treatment with CPT. B) Quantification of A, 3 biological replicates run in triplicate. * $P < 0.05$, ** $P < 0.01$, *** $P < 0.001$, **** $P < 0.0001$, one-way ANOVA with Welch correction. C) Quantification of APBs following CPT treatment in ATRX/DAXX knockout clones, > 200 nuclei analysed across 3 biological replicates. ** $P < 0.01$, **** $P < 0.0001$, one-way ANOVA with Welch correction.

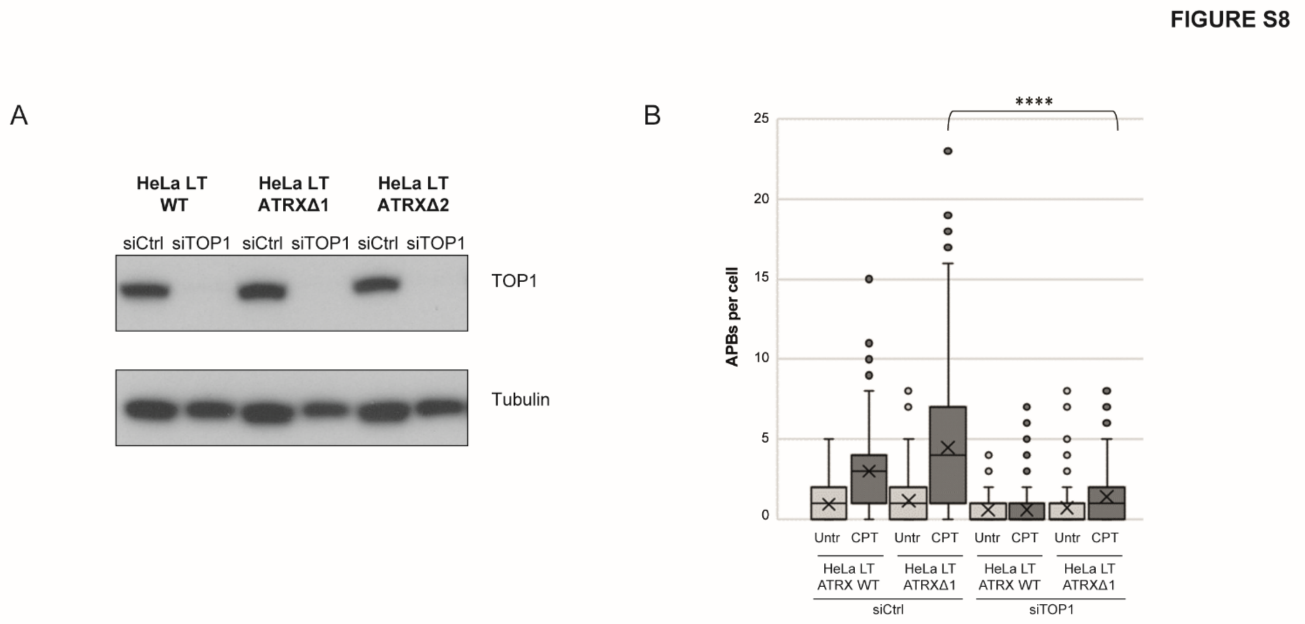

Supplementary Figure S8. TOP1 knockdown in CPT treated HeLa LT ATRX knockout cells diminishes APBs. A) Immunoblot of HeLa LT WT, ATRXΔ1 and ATRXΔ2 cells treated with siCtrl and siTOP1. B) Quantification of APB induction in HeLa LT ATRX knockout clones upon treatment with CPT, with and without siTOP1 knockdown, >200 nuclei analysed across 3 biological replicates. **** $P < 0.0001$, Kruskall-Wallis Test.

FIGURE S9

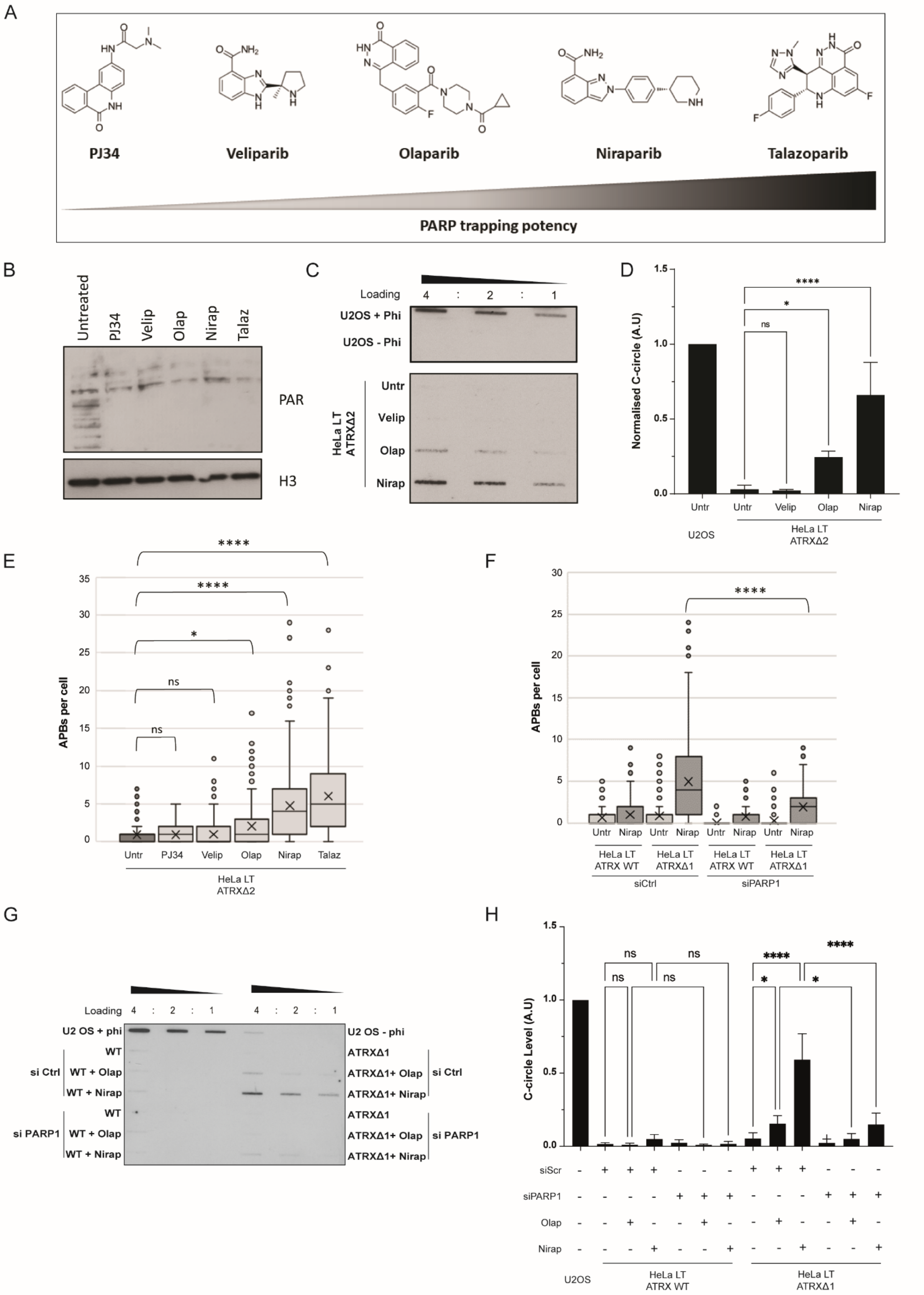
A
PJ34
Veliparib
Olaparib
Niraparib
Talazoparib
PARP trapping potency
B
Untreated
PJ34
Velip
Olap
Nirap
Talaz
PAR
H3
C
Loading 4 : 2 : 1
U2OS + Phi
U2OS - Phi
HeLa LT ATRXΔ2
Untr
Velip
Olap
Nirap
D
Normalised C-circle (A.U)
****
*
ns
Untr
Untr
Velip
Olap
Nirap
U2OS
HeLa LT ATRXΔ2
E
APBs per cell
****
****
*
ns
ns
Untr
PJ34
Velip
Olap
Nirap
Talaz
HeLa LT ATRXΔ2
F
APBs per cell
****
Untr
Nirap
HeLa LT ATRX WT
HeLa LT ATRXΔ1
siCtrl
siPARP1
G
Loading 4 : 2 : 1 4 : 2 : 1
U2 OS + phi
WT
WT + Olap
WT + Nirap
si Ctrl
si PARP1
U2 OS - phi
ATRXΔ1
ATRXΔ1+ Olap
ATRXΔ1+ Nirap
H
C-circle Level (A.U)
ns
****
*
siScr
siPARP1
Olap
Nirap
U2OS
HeLa LT ATRX WT
HeLa LT ATRXΔ1

Supplementary Figure S9. Trapping PARPi drugs lead to ALT induction in ATRX knockout cells and this is reversed by PARP1 knockdown. A) Schematic showing the structure and reported trapping potency of various PARPi drugs. B) Immunoblot showing PARylation levels in HeLa LT ATRXΔ1 cells following treatment with a panel of PARPi drugs for 48h at 5 μM. C) Representative C-circle blot showing induction of C-circles in HeLa LT ATRXΔ2 cells treated with trapping PARPi drugs. D) Quantification of C, 3 biological replicates run in triplicate. * $P < 0.05$, **** $P < 0.0001$, one-way ANOVA with Welch correction. E) Quantification of APBs in HeLa LT ATRXΔ2 following treatment with PARPi. * $P < 0.05$, **** $P < 0.0001$, Kruskall-Wallis Test. F) Quantification of APBs following niraparib treatment in the presence of siCtrl or siPARP1 knockdown. **** $P < 0.0001$, Kruskall-Wallis Test. G) Representative C-circle blot in olaparib and niraparib treated HeLa LT ATRX WT and ATRXΔ1 cells with and without siPARP1 knockdown. H) Quantification of G, 2 biological replicates run in triplicate. * $P < 0.05$, **** $P < 0.0001$, one-way ANOVA with Welch correction.

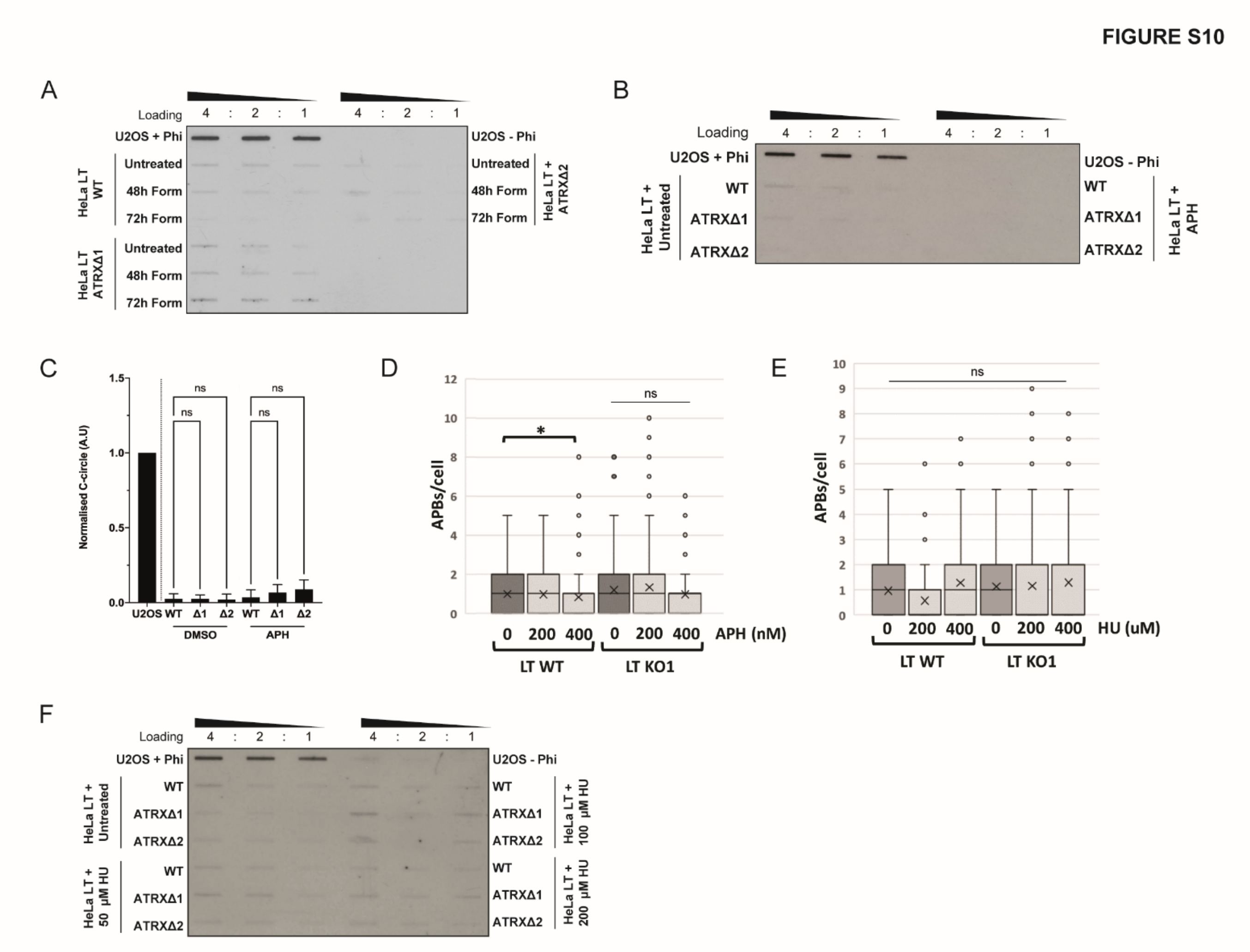

Supplementary Figure S10. Aphidicolin and Hydroxyurea fail to induce ALT hallmarks in combination with ATRX loss. A) C-circle blot showing that treatment of HeLa LT ATRX knockout cells fails to induce C-circle accumulation. Cells were treated with 500 µM formaldehyde and allowed to recover for either 48 hours or 72 hours. B) Representative C-circle blot in ATRX knockout clones following treatment with aphidicolin C) Quantification of B, 3 biological replicates run in triplicate. one-way ANOVA with Welch correction. D) Quantification of APBs in HeLa LT ATRX knockout clones upon treatment with low and high doses of aphidicolin, > 200 cells analysed across 3 biological replicates. Kruskall-Wallis Test. E) Quantification of APBs in HeLa LT ATRX knockout clones upon treatment with low and high doses of hydroxyurea, > 200 cells analysed across 3 biological replicates. Kruskall-Wallis Test. F) Representative C-circle blot of HeLa LT ATRX knockout cells treated with increasing doses of HU.

FIGURE S11

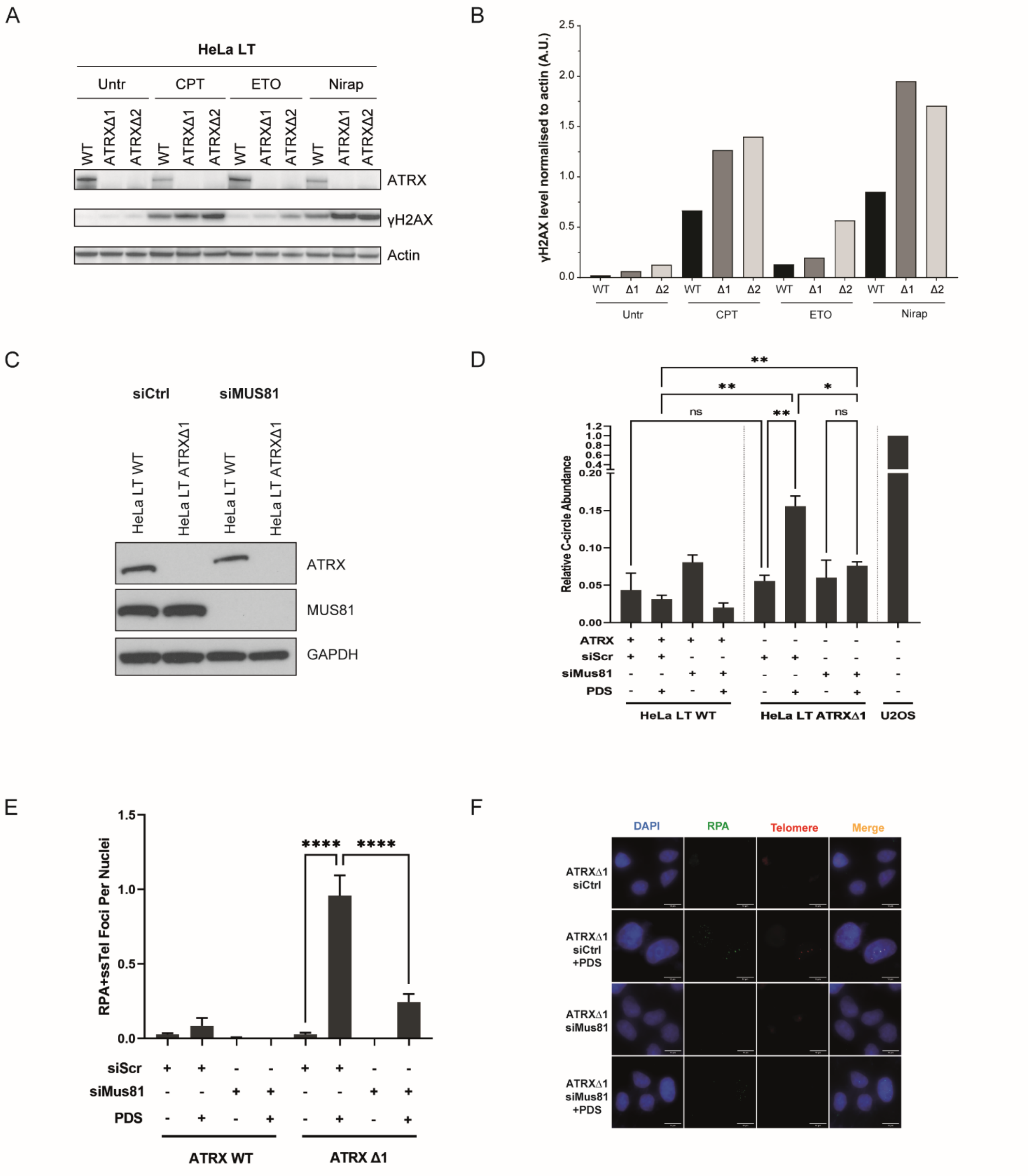
A
HeLa LT
Untr
CPT
ETO
Nirap
WT
ATRXΔ1
ATRXΔ2
ATRX
γH2AX
Actin
B
γH2AX level normalised to actin (A.U.)
2.5
2.0
1.5
1.0
0.5
0.0
WT
Δ1
Δ2
Untr
CPT
ETO
Nirap
C
siCtrl
siMUS81
HeLa LT WT
HeLa LT ATRXΔ1
ATRX
MUS81
GAPDH
D
Relative C-circle Abundance
1.2
1.0
0.8
0.6
0.4
0.20
0.15
0.10
0.05
0.00
**
*
ns
ATRX
siScr
siMus81
PDS
HeLa LT WT
HeLa LT ATRXΔ1
U2OS
E
RPA+ssTel Foci Per Nuclei
1.5
1.0
0.5
0.0
****
siScr
siMus81
PDS
ATRX WT
ATRX Δ1
F
DAPI
RPA
Telomere
Merge
ATRXΔ1 siCtrl
ATRXΔ1 siCtrl +PDS
ATRXΔ1 siMus81
ATRXΔ1 siMus81 +PDS

Supplementary Figure S11. PDS induced ALT markers require MUS81. A) Immunoblot showing increased levels of γH2AX in ATRX knockout clones treated with trapping agents. B) Quantification of A, γH2AX normalized to actin. C) Immunoblot showing siRNA mediated knockdown of MUS81 in ATRX WT and ATRX knockout cells. D) C-circle quantification showing induction of C-circles upon ATRX loss and PDS treatment is dependent on MUS81, 3 biological replicates run in triplicate. * $P < 0.05$, ** $P < 0.001$, one-way ANOVA with Welch correction. E) Quantification of immunoFISH data showing the induction of RPA ssTel foci upon ATRX loss and PDS treatment is dependent on MUS81, > 150 nuclei analysed across 3 biological replicates. **** $P < 0.0001$, one-way ANOVA with Welch correction. F) Representative RPA ssTel immunoFISH images.

FIGURE S12

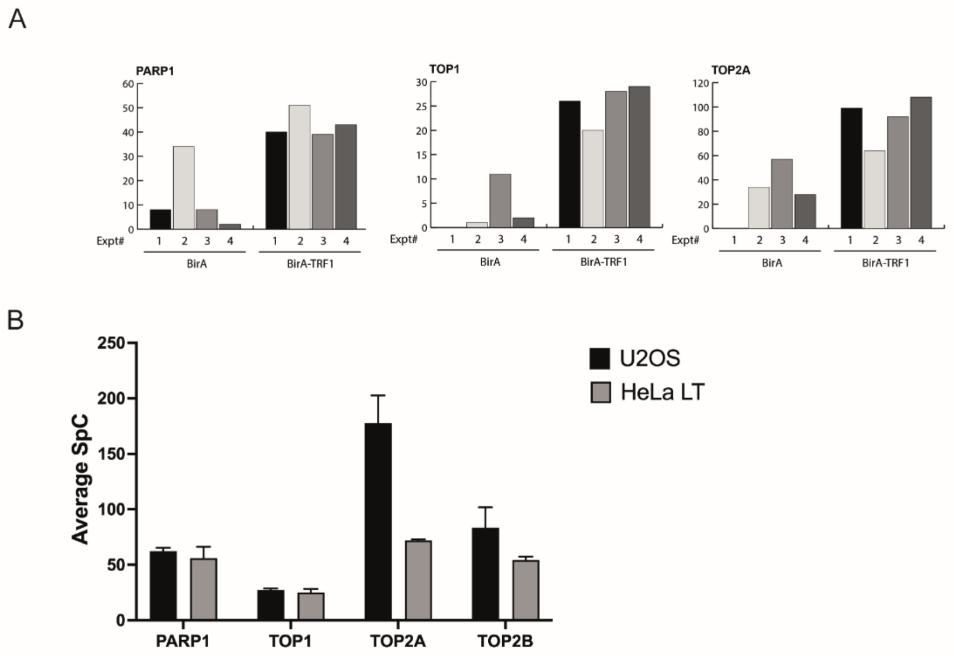

Supplementary Figure S12. Analysis of protein repertoire at telomeres by BioID. A) BioID proximity labelling data showed enrichment of PARP1, TOP1 and TOP2A at telomeric sequence in the U2OS cell line. BirA is a mutant biotin ligase (BirA Arg118Gly) which is fused to a protein of interest. Experiments were performed in quadruplicate. B) Comparison of U2OS and HeLa LT cell lines showed relative enrichment of TOP2A in the former. SpC = Spectral Counts.

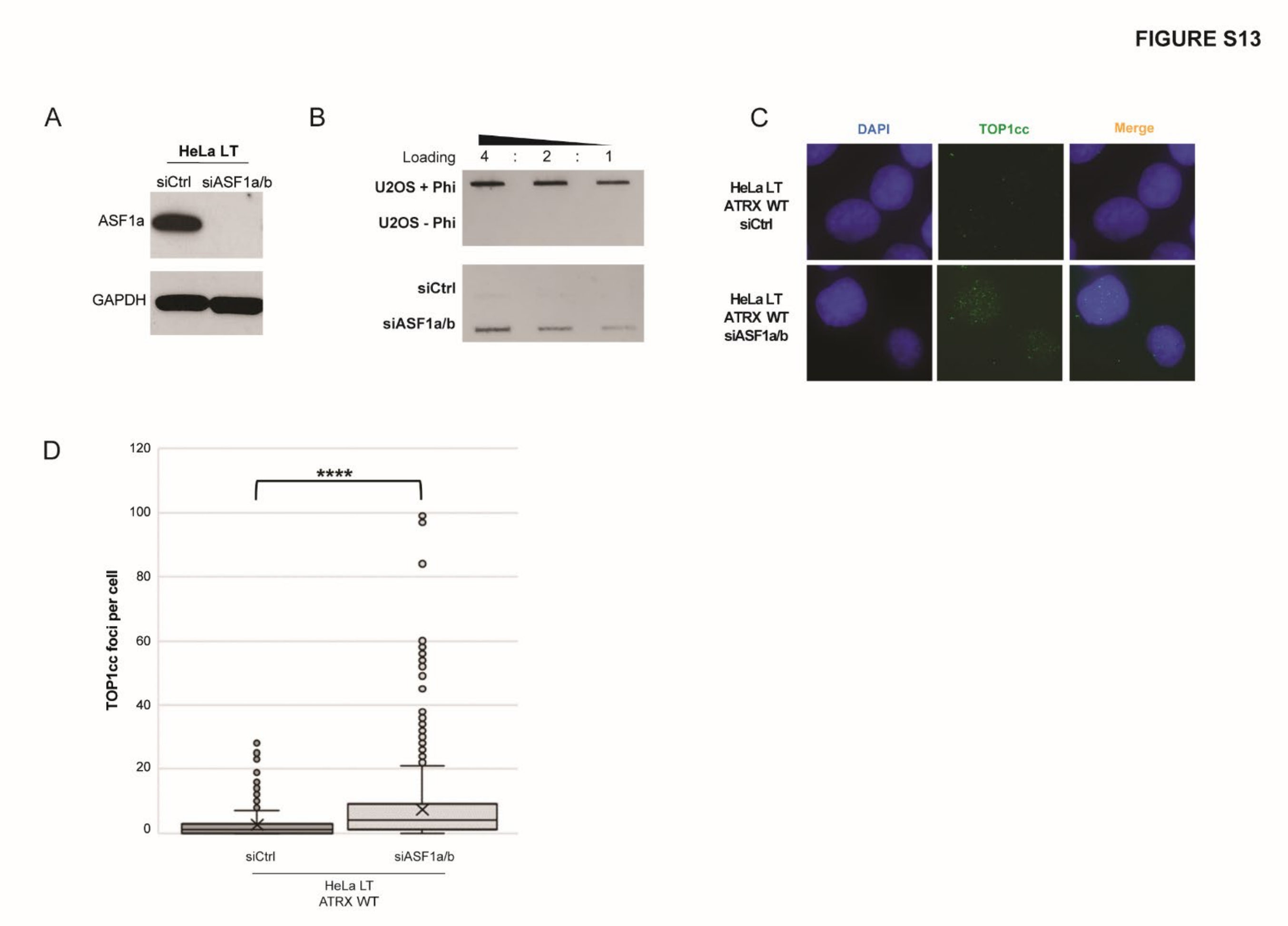

Supplementary Figure S13. ASF1a/b knockdown in HeLa LT cells leads to increased levels of trapped TOP1. A) Immunoblot of HeLa LT WT cells treated with siASF1a/b. B) C-circle blot showing induction of C-circles following ASF1a/b knockdown in HeLa LT cells. C) Representative immunofluorescence images of TOP1cc foci in siCtrl and siASF1a/b treated HeLa LT cells. D) Quantification of C, > 200 nuclei analysed across 2 biological replicates. **** $P < 0.0001$, Kruskall-Wallis Test.

FIGURE S14

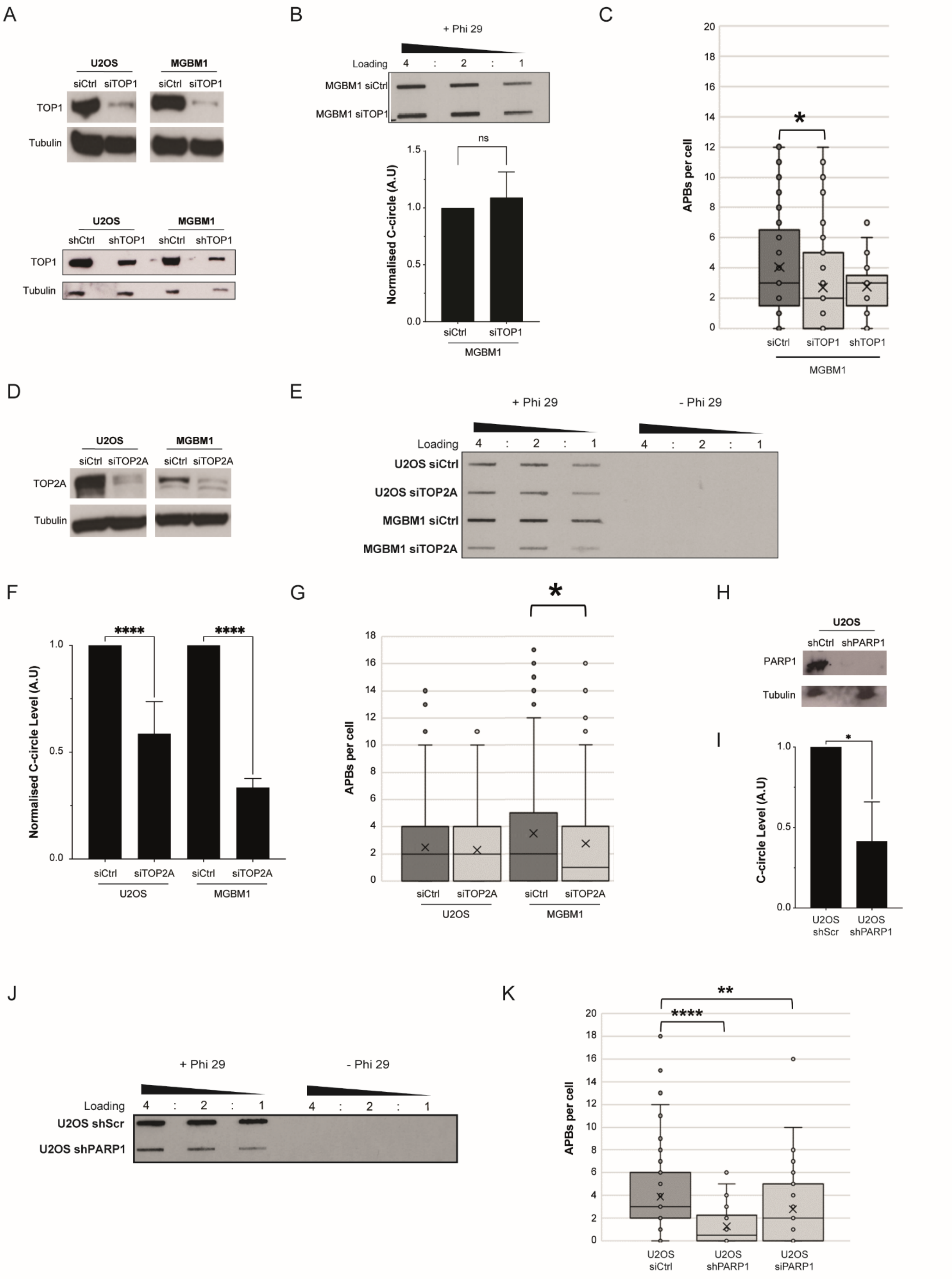

A
U2OS
MGBM1
siCtrl siTOP1 siCtrl siTOP1
TOP1
Tubulin
U2OS
MGBM1
shCtrl shTOP1 shCtrl shTOP1
TOP1
Tubulin
B
+ Phi 29
Loading 4 : 2 : 1
MGBM1 siCtrl
MGBM1 siTOP1
ns
Normalised C-circle (A.U)
siCtrl siTOP1
MGBM1
C
APBs per cell
siCtrl siTOP1 shTOP1
MGBM1
D
U2OS
MGBM1
siCtrl siTOP2A siCtrl siTOP2A
TOP2A
Tubulin
E
+ Phi 29
- Phi 29
Loading 4 : 2 : 1 4 : 2 : 1
U2OS siCtrl
U2OS siTOP2A
MGBM1 siCtrl
MGBM1 siTOP2A
F
Normalised C-circle Level (A.U)
siCtrl siTOP2A siCtrl siTOP2A
U2OS
MGBM1
G
APBs per cell
siCtrl siTOP2A siCtrl siTOP2A
U2OS
MGBM1
H
U2OS
shCtrl shPARP1
PARP1
Tubulin
I
C-circle Level (A.U)
U2OS shScr
U2OS shPARP1
J
+ Phi 29
- Phi 29
Loading 4 : 2 : 1 4 : 2 : 1
U2OS shScr
U2OS shPARP1
K
APBs per cell
U2OS siCtrl
U2OS shPARP1
U2OS siPARP1

Supplementary Figure S14. Knockdown of TOP1, TOP2A and PARP1 in ALT cancer cell lines diminishes ALT hallmarks. A) Immunoblots showing knockdown of TOP1 by siRNA and shRNA in U2OS and MGBM1 cells. B) Representative C-circle blot and quantification of C-circle levels in MGBM1 cells with siTOP1 knockdown, 3 biological replicates run in triplicate, unpaired t-test. C) Quantification of APBs in MGBM1 cells with siTOP1 and shTOP1 knockdown, > 200 nuclei analysed across 2 biological replicates. * $P < 0.05$, Kruskall-Wallis Test. D) Immunoblot showing knockdown of TOP2A by siRNA in U2OS and MGBM1 cells. E) Representative C-circle blot of U2OS and MGBM1 cells with TOP2A knockdown. F) Quantification of E, 3 biological replicates run in triplicate. **** $P < 0.0001$, one-way ANOVA with Welch correction. G) Quantification of APBs in U2OS and MGBM1 cells with siTOP2A knockdown, > 200 cells analysed across 3 biological replicates. * $P < 0.05$, Kruskall-Wallis Test. H) Immunoblot showing knockdown of PARP1 by shRNA in U2OS cells. I) Quantification of C-circle levels in U2OS cells treated with shPARP1, 2 biological replicates run in triplicate. * $P < 0.05$, unpaired t-test. J) Representative C-circle blot of shPARP1 knockdown in U2OS cells. K) Quantification of APBs in U2OS cells following PARP1 knockdown by siRNA and shRNA. > 200 cells analysed across 3 biological replicates. ** $P < 0.01$, **** $P < 0.0001$, Kruskall-Wallis Test.
